# *Drosera rotundifolia* L. as *E. coli* biofilm inhibitor: Insights into the mechanism of action using proteomics/metabolomics and toxicity studies

**DOI:** 10.1016/j.bioflm.2025.100268

**Published:** 2025-02-28

**Authors:** Sandy Gerschler, Sandra Maaß, Philip Gerth, Lukas Schulig, Toni Wildgrube, Jan Rockstroh, Martina Wurster, Karen Methling, Dörte Becher, Michael Lalk, Christian Schulze, Sebastian Guenther, Nadin Schultze

**Affiliations:** a Institute of Pharmacy, University of Greifswald, Friedrich-Ludwig-Jahn-Straße 17, 17489, Greifswald, Germany; b Institute of Microbiology, University of Greifswald, Felix-Hausdorff-Straße 8, 17489, Greifswald, Germany; c Institute of Biochemistry, University of Greifswald, Felix-Hausdorff-Straße 4, 17489, Greifswald, Germany; d Partner in the Greifswald Mire Centre, Soldmannstr. 15, 17487 Greifswald, Germany

**Keywords:** *Drosera rotundifolia*, Flavonoids, Naphthoquinones, Biofilm inhibition, Cytotoxicity, 3D cell culture model

## Abstract

The successful sustainable cultivation of the well-known medicinal plant sundew on rewetted peatlands not only leads to the preservation of natural populations, but also provides a basis for the sustainable pharmaceutical use of the plant. The bioactive compounds of sundew, flavonoids and naphthoquinones, show biofilm-inhibiting properties against multidrug-resistant, ESBL-producing *E. coli* strains and open up new therapeutic possibilities.

This study investigates the molecular mechanisms of these compounds in biofilm inhibition through proteomic analyses. Specific fractions of flavonoids and naphthoquinones, as well as individual substances like 7-methyljuglone and 2″-*O*-galloylhyperoside, are analyzed. Results show that naphthoquinones appear to act via central regulatory proteins such as OmpR and alter the stress response while flavonoids likely affect biofilm formation by creating an iron-poor environment through iron complexation and additionally influence polyamine balance, reducing intracellular spermidine levels. Further investigations including assays for iron complexation and analysis of polyamines confirmed the proteomic data. Safety evaluations through cytotoxicity tests in 3D cell cultures and the *Galleria mellonella in vivo* model confirm the safety of the extracts used. These findings highlight sundew as a promising candidate for new phytopharmaceuticals.

## Highlights

• Flavonoids and naphthoquinones from *Drosera* inhibit biofilm formation in ESBL-*E. coli.*
• Proteome and metabolome analyses suggest a potential mechanism of action.
• Flavonoids may inhibit biofilm formation through iron chelation and polyamine balance.
• Naphthoquinones show effects on stress response pathways and biofilm regulatory point OmpR.
• In vitro toxicity tests and *Galleria mellonella* model confirm the safety of *Drosera* extracts.

## 1 Introduction

Traditionally, sundew (*Drosera* species pluralis [spp.]) has been used in Central Europe to treat spasmodic respiratory infections. However, despite their known effectiveness, sundew is no longer part of modern phytotherapy practices anymore. Due to unsustainable wild harvesting and destruction of peatlands over decades, sundew is a protected species in most European countries. Experiments with growing *Drosera* sp. on peat mosses in northern Germany have shown that it is now possible to cultivate European species, making sundew not only commercially available but also contributing to the sustainable use of peatlands. Peatlands represent significant ecosystems that play a fundamental role in the global carbon cycle and thus in climate protection. In recent years, the rewetting of peatlands has gained increasing attention and implementation, driven by the growing awareness of their ecological significance and potential as habitats for climate and biodiversity protection [1]. Despite the ecological benefits of peatland rewetting, economic challenges need to be addressed, particularly with regard to potential conflicts with agricultural land. It is therefore essential to develop incentive systems that support the sustainable use and protection of these sensitive habitats is essential.

The potential economic importance of sundew lies primarily in its use as a medicinal plant for the production of phytopharmaceuticals or, after extraction, therapeutically used single compounds. One challenge is the intrinsic variability of plant compounds, which is influenced by environmental factors such as soil composition, precipitation patterns, and climatic conditions. In addition, variations of compound concentrations that determine the effectiveness can lead to over- or underdosing and thus endanger the patient. Therefore, quality parameters and testing procedures for sundew as a medicinal plant have recently been published. These test methods include not only quantitative content analysis, but also techniques to distinguish the round-leaved sundew (*Drosera* [*D.*] *rotundifolia* L.) from other European species, such as the middle sundew (*D*. *intermedia* Hayne) [2]. Recent studies have shown while the qualitative composition of the active compounds may be similar within the European species, they differ quantitatively. This distinction is crucial, especially considering our previous exploration of a new application for sundew as virulence blocker due to its biofilm inhibiting activities. This investigation of sundew (*Drosera* spp.) revealed biofilm inhibitory properties against biofilm-forming multidrug-resistant *E. coli* strains [3]. *E. coli* is a common gut inhabitant of birds and mammals, including humans. Due to its readily accessible natural and random genetic changes, it is a versatile bacterial species consisting of commensal and pathogenic subtypes [4,5]. Uropathogenic *E. coli* (UPEC) are among the most common causes of urinary tract infections, leading to more than 400 million cases in 2019 [6]. Some *E. coli* strains can produce enzymes such as Extended spectrum beta-lactamases that hydrolyze and thus inactivate antibiotics [7]. In addition, certain strains are not only able to maintain their multidrug-resistance without antibiotic treatment, but also combine it with virulence characteristics such as biofilm formation [8]. The combination of resistance and virulence has led to the pandemic spread of such strains. In a phenotypic assay, the impact of *D. rotundifolia* L. on the formation of curli and cellulose, two main components of the extracellular matrix of the biofilm, was examined [3]. Not only a mixture of various sundew compounds in extracts was tested but also enriched fractions and single substances. It was found that flavonoids, including 2″-*O*-galloylhyperoside, are particularly important for the biofilm-inhibiting properties of sundew. As sustainable cultivation seems possible, *D. rotundifolia* L. could play a crucial role in the future in both the prevention and treatment of recurrent infections associated with the biofilm formation by UPEC strains [3].

This study aims to evaluate the toxic potential of *D*. *rotundifolia* L. and at the same time to investigate its pharmaceutical potential by analyzing a possible molecular mechanism of its biofilm inhibitory effect. Caution is advised especially regarding the naphthoquinone content of the different sundew species, as naphthoquinones such as plumbagin and 7-methyljuglone are known for cytotoxic effects [2,9]. However, the previous study used monolayer cell cultures for the investigation. Given ethical concerns about animal experiments and species-specific differences, using human cell cultures is still the method of choice in research and development despite known limitations. Monolayer cultures tend to overestimate toxicity as they do not reflect the complexity of human tissues [10]. Three-dimensional (3D) cell models are often used to overcome this problem. Therefore, this study used a 3D model based on TR146 cells as well as the *in* *vivo Galleria mellonella* model to evaluate the toxic potential of *D*. *rotundifolia* L. in detail.

It is still unclear what effect naphthoquinones and flavonoids have on the biofilm of *E. coli* and how they might differ, or whether synergistic effects of both compound classes play a role. Based on a phenotypic macrocolony assay, proteomic and metabolomic analyses were carried out on *D. rotundifolia* L. samples to gain insights into the mechanisms of action. These analyses included extracts, fractions and single compounds. In both examined fractions of *D*. *rotundifolia* L., the two main groups of active compounds, flavonoids, and naphthoquinones, were enriched respectively to investigate the effects of these compound groups. In parallel, an important single compound representative of each class was analyzed individually to gain targeted insights into their effects on protein homeostasis. The proteome and metabolome analyses indicated that the polyamine pathway is a possible target. Therefore, *in* *silico* docking studies and intracellular quantification of spermidine and putrescine, two polyamines with known impact on biofilm formation were carried out. Also, the iron-binding capacity of *D. rotundifolia* L. samples was analyzed, as the availability of iron plays a decisive role for virulence factors and, thus, the infection process.

## 2 Results and discussion

### 2.1 Evaluation of toxicity

In a previous study the biofilm of multidrug-resistant *E. coli* was proven to be a target of sundew in particular of *D*. *rotundifolia* L. Research in modern phytotherapy should not just focus on new applications of natural products but also prove that they are not toxic and safe.

**Fig. 1:**
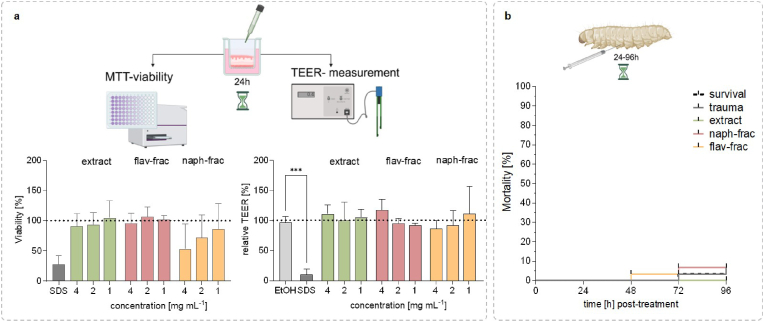
Evaluation of toxic effects by different treatments (*D*. *rotundifolia* L. extract, naphthoquinones- and flavonoids-fractions). **(a)** 3D mucosa equivalents were treated with various concentrations for 24 h. Viability was assessed with MTT assay, and TEER was determined. Relative TEER is the value t:24 h in relation to t:0 h. 2.5% SDS served as positive control. 2% EtOH served as a vehicle. The dotted line represents the 100% viability of the vehicle treated cells. mean + SD; n = 3. One-way ANOVA post hoc Dunnett's test ∗∗∗p< (0.0001) **(b)***Galleria mellonella* larvae were treated with 2000 mg kg^−1^ (n = 3). Results are shown as Kaplan–Meier plot of mortality, created with BioRender.com.

In the same study, extracts of different *Drosera* spp. were examined, showing cytotoxic effects on two monolayer cell lines (TR146 and A549) cultures in a range of 6–65 μg mL^−1^ which is given as the IC_50_ (=the concentration of an inhibitor at which half-maximal inhibition is observed) [2]. Although monolayer cultures are pivotal in many aspects, they tend to under- or overestimate toxicity as they do not adequately represent the complexity of human tissue [[11], [12], [13]]. Therefore, this study used a complex 3D mucosa model consisting of several cell layers. The cell viability of the 3D mucosa models was not affected after treatment with extract and the flavonoid-fraction. A concentration-dependent decrease in viability is seen under treatment with the naphthoquinone fraction. This fraction was enriched in, 7-methyljuglone and plumbagin, well known for their cytotoxic effects [2,9]. The relative TEER (transepithelial electrical resistance) value did not change significantly compared to the EtOH treated controls, indicating that cell integrity was maintained under all treatments (Fig. 1a).

A further possibility to overcome the limitations of cell culture-based methods is the use of *Galleria mellonella* (wax moth) larvae as a non-vertebrate test system to determine acute toxicity [[14], [15], [16]]. Acute toxicity refers, to the adverse effects that may occur after ingestion (oral or dermal) of a single dose of a substance or multiple doses within 24 h. Here, extracts and enriched fractions were tested in a single dose of 2000 mg kg^−1^ and the mortality was recorded daily for four days. Within the first 24 h, none of the larvae died from the treatment. Overall, the mortality rate was very low during the investigated period (Fig. 1b). One larva died after 48 h of treatment with the flavonoid fraction, two deaths were recorded after 72 h of treatment with the naphthoquinone fractions, respectively. Unfortunately, the control groups also had two cases of deaths after 72–96 h. Therefore, the observed mortality under treatment might be due to a general indisposition of the larvae as they were not fed during the experiment. The Globally Harmonised System (GHS) categorizes chemicals into a system based on the lethal dose values from low toxicity (category 5, 2000–5000 mg kg^−1^) to high toxicity (GHS category 1; <5 mg kg^−1^). If the data obtained are assigned to these categories, the extract and the fractions would probably be category 4 (300- ≤ 200 mg kg^−1^) as the flavonoid rutin and therefore have a low toxicity.

### 2.2 Analysis of mechanism of action against biofilm formation

Biofilm-associated infections complicate therapy and often lead to recurrences [17,18]. Active compounds that influence the formation and composition of biofilms can reduce the pathogenicity of such infectious agents and improve therapeutic success. A central approach is the inhibition of extracellular matrix formation, a crucial process in biofilm growth. In *E. coli*, this matrix mainly consists of amyloid fibers (curli) and exopolysaccharides, especially cellulose, β-1,6-*N*-acetyl-d-glucosamine, and colanic acid [19]. A recently published study investigated the phenotypic influence of *D*. *rotundifolia* L. on the formation of curli and cellulose during matrix development in three multidrug-resistant *E. coli* strains, including the globally prevalent and antibiotic-resistant ST131 lineage. The results showed that compounds from this plant could inhibit the production of the extracellular matrix in *E. coli* ST131 [3].

In this study, proteomic analyses were performed on *E. coli* ST131 macrocolonies that had been incubated with various *D*. *rotundifolia* L. extracts under biofilm-forming conditions. Changes in protein abundance were analyzed under the influence of different *D*. *rotundifolia* L. extracts, compared to untreated *E. coli* ST131 macrocolonies incubated under identical conditions. The extracts included a *D*. *rotundifolia* L. whole extract, two compound enriched fractions and two pure compounds. The fractions were enriched with the two main active compound groups of *D*. *rotundifolia* L.: flavonoids and naphthoquinones. The pure compounds included the flavonoid 2″-*O*-galloylhyperoside and the naphthoquinone 7-methyljuglone, both main constituents of *D*. *rotundifolia* L. The aim of the investigation was to examine the effects of various *D*. *rotundifolia* L. extracts and pure compounds on the proteome of *E. coli* ST131 during biofilm formation.

In the first step, the data (available in the database on the ENA server [PBIO729=IMT17433 = ERR163891=Sanger Cambridge Number 8016_2_57, downloaded October 10, 2022, 4885 entries]) were analyzed purely quantitatively by determining the number of proteins whose abundance was significantly altered in the respective treatments. The analysis showed a statistically significant change in the abundance of 146 proteins under the influence of *D*. *rotundifolia* L. compared to the untreated control (ANOVA, p < 0.05, n = 3). These proteins were distributed among the respective treatments as follows: 113 proteins were differentially abundant in the presence of the *D*. *rotundifolia* L. whole extract, 82 proteins were affected by the naphthoquinone enriched fraction, 103 proteins by the flavonoid enriched fraction, 71 proteins under the influence of 7-methyljuglone, and 64 proteins with 2″-*O*-galloylhyperoside.

**Fig. 2:**
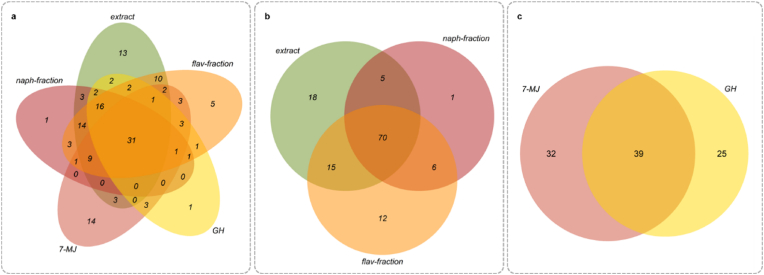
Venn diagram (created with R v4.2.2) of proteins whose abundance was significantly altered (Log2-FC > |0.8|, p < 0.05) in an *E*. *coli* (PBIO729) macrocolony following a 48 h incubation at 28 °C with various *D. rotundifolia* L. preparations. These samples include an extract, naphthoquinone (naph-fraction), and flavonoid fraction (flav-fraction), as well as single compounds such as 2″-*O-*galloylhyperoside (GH) and 7-methyljuglone (7-MJ).

In Fig. 2, the different *D*. *rotundifolia* L. preparations are represented as circles. The numbers within the intersecting areas indicate the number of proteins whose abundance was altered by multiple preparations. The number inside each circle indicates the number of proteins whose abundance was specifically affected by a particular treatment. Comparing all treatments shows that most of the proteins are found in the overlapping areas. When comparing all treatments, the abundance of only one protein was changed solely by treatment with 2″-*O*-galloylhyperoside or the naphthoquinone fraction. The abundance of five proteins was modified by the flavonoid fraction, 13 proteins by the extract treatment, and 14 proteins by 7-methyljuglone (Fig. 2a). This trend is even more pronounced when comparing only the crude extract to the two different enriched fractions (Fig. 2b), showing that the abundance of 70 proteins was influenced across all three different treatments. The abundance of one protein was specifically influenced by the naphthoquinone fraction, 12 proteins by the flavonoid fraction, and 18 proteins exclusively by the extract treatment. When compared, the single compounds, 7-methyljuglone and 2″-*O*-galloylhyperoside, showed a slightly different pattern (Fig. 2c). Here, the number of proteins within the shared intersecting area (39) is comparable to the individual areas of the two substances (32 proteins for 7-methyljuglone, 25 proteins for 2″-*O*-galloylhyperoside).

One reason for the high number of proteins within the intersecting areas could be that the treatments affect similar molecular signaling pathways and the enriched fractions are not pure single compounds.

To gain insights into effected biological signaling pathways, proteins with significantly altered abundance were categorized into functionally similar groups (Fig. S1). The analysis revealed a consistent clustering pattern across all treatments. However, the targeted enrichment of naphthoquinones and flavonoids in the respective fractions did not show a specific clustering pattern indicative of different mechanisms of action. It is possible that the complex composition of the fractions leads to overarching activity patterns rather than clearly differentiating the specific effects of the compound groups. Also, a more general stress response could be a potential explanation. Additionally, the observed effects may result from the interaction of multiple target structures. Substances like flavonoids and naphthoquinones can induce various types of stress in microorganisms like *E. coli*, depending on their chemical properties and concentrations [[20], [21], [22]]. Possible interactions might include enzyme inhibition or changes to the cell membrane, leading to disturbances in energy metabolism or protein synthesis, which is referred to as metabolic stress. Furthermore, oxidative stress could also occur [23,24]. Although flavonoids generally possess antioxidant properties, high concentrations during metabolism or direct interactions with oxidative systems in the bacterium might lead to the formation of reactive oxygen species [25,26]. A qualitatively similar pattern was observed in the case of the single substances (Fig. S1c), albeit with quantitative variations. For example, 2″-*O*-galloylhyperoside was associated with a higher number of proteins involved in transport processes and cofactor biosynthesis, while 7-methyljuglone was more frequently associated with proteins involved in amino acid metabolism.

**Fig. 3:**
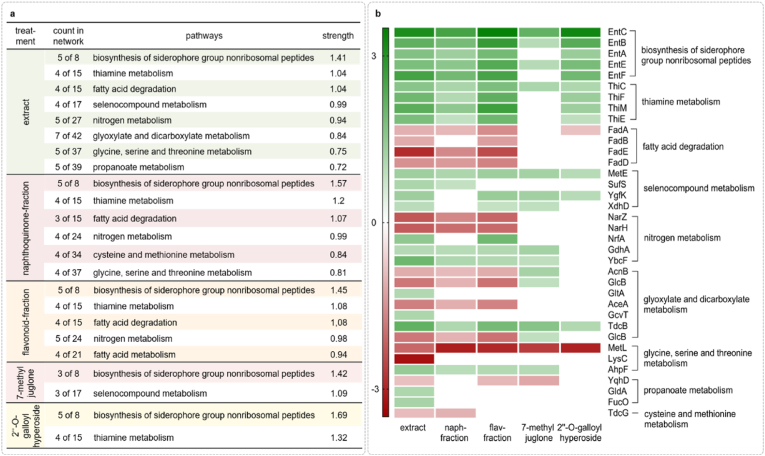
**(a)** Regulated metabolic/KEGG pathways based on significantly altered protein abundances by the treatments (*D*. *rotundifolia* L. crude extract, naphthoquinones- and flavonoids-fractions, 7-methyljuglone and 2″-*O*-galloylhyperoside) (Log2-FC > |0.8|, p < 0.05). The pathways were identified using the STRING v12.0 database (*E. coli* K12). "Count in network" indicates the number of proteins per pathway, "strength" shows the enrichment ratio. Strength indicates how much more frequently proteins in a network are annotated with a term compared to what would be expected by chance. (**b**) Heatmap of the altered protein abundances (Log2-FC) in the identified metabolic pathways under the various treatments: *D*. *rotundifolia* L. extract, naphthoquinone fraction, flavonoid fraction, 7-methyljuglone, and 2″-*O*-galloylhyperoside. The color scale ranges from red (decreased abundance) to white (no change) to green (increased abundance).

A STRING analysis (https://string-db.org/) was conducted to comprehensively analyze the biological functions and interactions of differentially expressed proteins. This analysis enables the identification of protein-protein interactions and the assignment of proteins to specific metabolic pathways. Fig. 3a presents the most relevant metabolic pathways that showed a strength of Log10 ≥ 0.7. This score indicates the ratio of the number of proteins in a network annotated with a term to the number of proteins expected to be annotated with that term in a randomly generated network of the same size. Additional details, including locus tags, protein names and functions of selected proteins, are summarized in Table S5. These pathways were identified using the STRING database, version 12.0, for *E. coli* K12. In Fig. 3b, the protein abundance assigned to a metabolic pathway under the *D*. *rotundifolia* L. treatments is visualized through a color pattern, where a red coloration indicates a decrease and a green coloration indicates an increase in protein abundance. Five out of eight proteins in the metabolic pathway of non-ribosomal peptide biosynthesis of the siderophore group showed increased protein amounts under the treatment conditions of the crude extract, the naphthoquinone fraction, the flavonoid fraction, and 2″-*O*-galloylhyperoside. The treatment with 7-methyljuglone resulted in differential abundance of only three out of eight proteins. This observation could indicate an enhanced production of siderophores, which are synthesized by bacteria for iron uptake. This increased synthesis might be a response to stress conditions induced by the treatments. Another significant metabolic pathway is thiamine biosynthesis, where treatments with the extract, the fractions, and 2″-*O*-galloylhyperoside showed changes in abundance in four out of 15 proteins. Thiamine (vitamin B1) is an essential cofactor in a variety of enzymatic reactions and plays a central role in energy and carbohydrate metabolism. The altered abundance of proteins within this pathway could suggest that the treatments influence the cellular energy metabolism. Four out of 15 proteins involved in fatty acid degradation showed altered abundance due to the treatment with the extract and the flavonoid fraction, while three out of 15 proteins were affected by the naphthoquinone fraction. The single compounds 7-methyljuglone and 2″-*O*-galloylhyperoside yielded little or no changes. The observed effects on fatty acid metabolism could also indicate synergistic interactions between different compounds within the extract or fractions. Such interactions may enhance or modify the activity of single compounds, leading to more pronounced changes in protein abundance than observed with isolated compounds like 7-methyljuglone or 2″-*O*-galloylhyperoside. This hypothesis is supported by the complex chemical composition of the *D. rotundifolia* L. extract, which includes various flavonoids and naphthoquinones that may act on multiple metabolic pathways simultaneously. A potential consequence of this influence on lipid metabolism could be alterations in cell membrane integrity and energy production [27].

In the course of the study, the most relevant metabolic pathways, e.g., siderophore biosynthesis and thiamine metabolism, were selected as a basis for in-depth analysis. Siderophores are small molecules synthesized by microorganisms to chelate iron from the environment and make it available for cellular metabolism. Iron is an essential nutrient required for numerous biological processes, including cellular respiration, DNA replication, and immune response [28]. Its role in biofilm formation varies among bacterial species; iron deficiency promotes biofilm formation in certain species, such as *Streptococcus mutans* [29], while inhibiting it in others like *Vibrio cholerae* [30]. Research has shown that iron restriction can reduce biofilm formation in *Pseudomonas aeruginosa* by blocking macrocolony initiation [31]. Similarly, iron appears pivotal in *E. coli* biofilm formation, influencing the transition from sessile to mobile forms under iron-limiting conditions. A study indicates that biofilm formation by *E. coli* is reduced under otherwise rich conditions due to iron limitation, with existing biofilms exhibiting sensitivity to such restrictions [32].

**Fig. 4:**
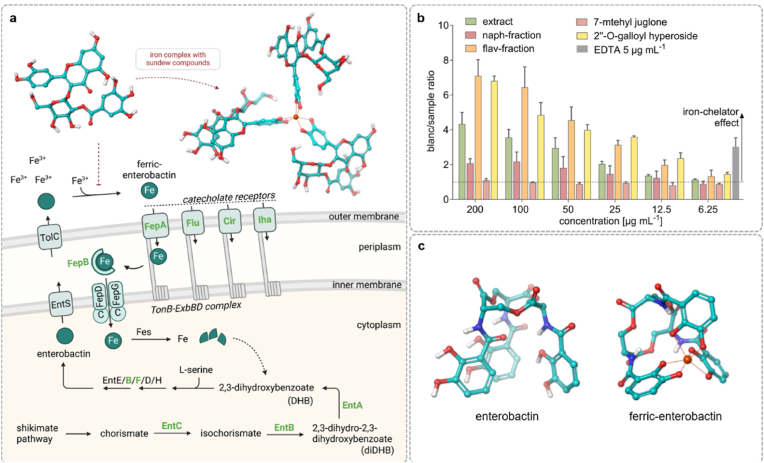
**(a)** Potential effects of *D*. *rotundifolia* L. on the iron balance in *E. coli* (PBIO729), with a focus on iron transport via enterobactin. Key elements include the production of enterobactin through the activity of enterobactin synthase (EntE/B/F/D/H). Enterobactin then forms a complex with iron (Fe), resulting in ferric-enterobactin, which is transported across the outer membrane via catecholate receptors (FepA, Flu, Cir, Iha, and IreA) and the TonB-ExbBD complex. In the periplasm, ferric-enterobactin is processed by Iron (III) enterobactin esterase (Fes), releasing free iron (Fe) that enters the cytoplasm, created with BioRender.com. **(b)** shows the results of the CAS assay on the iron complexing properties of different *D*. *rotundifolia* L. preparations (crude extract, naphthoquinone, and flavonoid fractions, 7-methyljuglone, and 2″-*O*-galloylhyperoside). The measured absorption of the blank was divided by the absorption of the samples. In the absence of chelators, the value is one; if a substance binds iron, this value increases*,* n = 3 mean +SD. **(c)** shows the structure of ferric-enterobactin and enterobactin, in the structural formula, the red spheres symbolize oxygen atoms, the white spheres hydrogen atoms and the dark blue spheres nitrogen atoms, the carbon backbone is shown in turquoise.

The potential iron limitation could be attributed to the ability of flavonoids [33,34] and naphthoquinones [35] to complex iron (III) ions, thereby hindering iron uptake by microorganisms. This resulting iron deficiency can negatively impact the growth and proliferation of *E. coli*, particularly within biofilm-forming communities. Indicators of iron deficiency found in our analysis include altered levels of proteins involved in enterobactin biosynthesis, such as EntA, EntB, EntC, and EntF [36] (Fig. 3b). Enterobactin is a siderophore that binds iron from the environment (Fig. 4c) and transports it into the cells (Fig. 4a). In addition to the biosynthesis proteins, an increased amount of transport proteins such as FepA, Fiu, Cir, and Iha was observed under the influence of *D. rotundifolia* L. (Fig. 4a). These proteins are catecholate receptors responsible for the uptake of iron bound by enterobactin. An iron deficiency could lead to enhanced expression of these transport proteins to both increase the production of enterobactin and improve iron uptake through increased transport capacity. This organization of iron binding and uptake is illustrated in Fig. 4a. To gather further evidence for this hypothesis, a Chrom-Azurol-S (CAS) assay was conducted. This test aimed to determine whether the *D. rotundifolia* L. preparations are capable of complexing iron. The samples were tested at concentrations ranging from 200 to 6.25 μg mL^−1^. The range was chosen based on the concentrations at which the *D. rotundifolia* L. preparations showed a biofilm-inhibitory effect (75–150 μg mL^−1^). The results showed that the flavonoid fraction, 2″-*O*-galloylhyperoside, and the extract exhibited iron-complexing properties to a concentration of 6.25 μg mL^−1^ (Fig. 4b). Quercetin, a well-researched and widely distributed flavonoid found in *D. rotundifolia* L., forms the structural basis for other flavonoids such as isoquercitrin and 2″-*O*-galloylhyperoside. It is known as a phytochelator capable of binding both Fe^2+^ and Fe^3+^ [37,38], suggesting that flavonoid-containing plant extracts in general could potentially influence the biofilm formation of *E. coli*. Notably, *D. rotundifolia* L. has a high flavonoid content (up to 5%) [39], with 2″-*O*-galloylhyperoside being one of its main flavonoids makes it an especially interesting plant for further study. In previous studies, 2″-*O*-galloylhyperoside demonstrated strong biofilm-inhibitory effects [3], which may be related to its exceptional iron-binding capacity. The iron-binding capability of 2″-*O*-galloylhyperoside can be attributed to numerous electron donor groups, which provide a variety of potential coordination sites for chelation. These manifold ligand sites enable the formation of stable metal-polyphenolic networks (MPN) in which multiple 2″-*O*-galloylhyperoside molecules simultaneously interact with iron ions, resulting in a robust multi-ligand complex. This cooperative binding increases the overall stability and maximizes the chelation potential [40,41]. The naphthoquinone fraction demonstrated iron-complexing activity up to 100 μg mL^−1^, while 7-methyljuglone showed only a slight iron-complexing effect at 200 μg mL^−1^ (Fig. 4b). The naphthoquinone fraction contains enriched naphthoquinones; however, a complete separation from flavonoids was not achieved, resulting in approximately 1% quercetin. Therefore, the iron-binding capacity of the naphthoquinone fraction could be attributed to the accompanying substance quercetin. The analysis of 7-methyljuglone did not reveal any specific influence on a signaling cascade. Still, it was interesting to note in the proteome analysis that only 7-methyljuglone influenced the abundance of the proteins UspE and OmpR. UspE is a protein expressed under stress conditions and contributes to overall stress resistance, while OmpR is a regulatory protein that is part of a two-component system and plays a central role in biofilm formation and iron uptake regulation [42,43]. This could explain why the treatment with 7-methyljuglone, which showed no complexation properties in the CAS assay, showed a regulation of the enterobactin synthesis pathway in proteome analysis. Overall, these findings support the hypothesis that *D. rotundifolia* L. preparations influence the iron homeostasis of *E. coli*.

The proteomic analysis also revealed an increased abundance of the proteins ThiC, ThiF, ThiM, and ThiN, which are involved in the thiamine metabolic pathway. The active form of vitamin B1, thiamine pyrophosphate (TPP), is an essential cofactor for several anabolic and catabolic reactions in central metabolism. TPP-dependent enzymes, including transketolase, pyruvate dehydrogenase, 2-oxoglutarate dehydrogenase, alpha-ketoglutarate dehydrogenase, and 1-deoxy-d-xylulose-5-phosphate synthase, catalyze key cellular processes in bacteria, ranging from central metabolism to the biosynthesis of amino acids, cofactors, and lipids [44]. An increased abundance of these proteins could indicate a metabolism altered by the *D. rotundifolia* L. preparations.

In addition to the proteome analysis, this study also examined the effects of the *D. rotundifolia* L. preparations on the metabolome of *E. coli* ST131. To investigate how metabolism changes during biofilm formation, targeted metabolome analysis of 63 metabolites was conducted after 24 and 48 h. *D. rotundifolia* L. preparations had little impact on the abundance of metabolites, only a few changes of individual metabolites involved in carbohydrate metabolism, the tricarboxylic acid cycle, amino acid metabolism, and fatty acid degradation were found. These changes were, in particular, evident after 24 h. The increased abundance of the metabolite citrate (2.5–6 fold), which has also been described as a siderophore in *E. coli* [45], is of special interest since it could be a compensatory metabolism of the iron depletion caused by *D*. *rotundifolia* L. preparations (Fig. S2 and Table S1-4, Supporting information).

In addition to iron homeostasis, polyamine equilibrium plays a crucial role in biofilm formation. Polyamines such as putrescine, spermidine, and spermine are small, positively charged organic compounds containing two or more amino groups. These compounds are present in both eukaryotic and prokaryotic cells and are involved in numerous cellular processes, including the stabilization of DNA, RNA, proteins, and cell membranes. Furthermore, they regulate ion transport and the membrane potential while also influencing various intracellular signaling pathways.

**Fig. 5:**
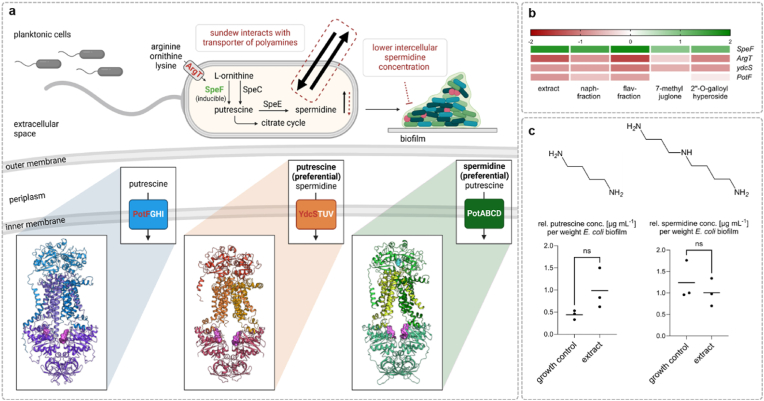
(**a**) Potential effects of *D. rotundifolia* L. on the polyamine (spermidine and putrescine) balance in *E*. *coli* PBIO729. It shows the biosynthesis pathway of spermidine, which is formed from ornithine via putrescine, catalyzed by the enzymes SpeC and SpeE. Putrescine can also be synthesized through the inducible enzyme SpeF. The depicted transport systems include PotFGHI, PotABCD, and YdcSTUV, which transport both spermidine and putrescine, created with BioRender.com. (**b**) Heatmap of the altered protein abundances in the identified metabolic pathways under the various treatments: *D. rotundifolia* L. extract, naphthoquinone fraction, flavonoid fraction, 7-methyljuglone and 2″-*O*-galloylhyperoside. The color scale ranges from red (decreased abundance) to white (no change) to green (increased abundance). (**c**) Putrescine concentration (left) and spermidine concentration (right) per 1 mg E. *coli* macrocolony incubated at 28 °C with *D. rotundifolia* L. extract and without (growth control) measured using LC-MS, n = 3 mean +SD. Unpaired *t*-test with Welch's correction (p < 0.05), ns = not significant.

*E. coli* can synthesize the polyamines spermidine, putrescine, and norspermidine. Spermidine is derived from putrescine, which is itself formed from ornithine or arginine, while cadaverine is synthesized from lysine. Apart from synthesizing polyamines, *E. coli* can also up take polyamines from the environment or export them. The transport of these molecules across the cell membrane is complex and mediated by specialized transport systems. These include ABC transporters such as the polyamine transport system (Pot-System), PotABCD and PotFGHI, which transport polyamines from the periplasm into the cytoplasm [46]. Additional transport mechanisms involve antiporters like CadB and importers like PuuP, which utilize proton gradients to transport putrescine into the cell. Furthermore, MdtJI multidrug efflux transporters remove both polyamines and other toxic substances from the cell [47] (Fig. 5). Maintaining cellular polyamine balance is critical for cell survival, as excessive concentrations can be toxic. An investigation into the effect of spermidine on biofilm formation in *E. coli* K-12 revealed that intracellular spermidine concentration promotes biofilm formation and that the spermidine transporter protein PotD is likely involved in this process [48].

Interestingly, our analysis showed a decrease in the abundance of proteins such as PotF, YdcS, and ArgT, along with an increase in the abundance of the protein SpeF under the influence of *D. rotundifolia* L. preparations (Fig. 5b). These proteins are involved in the transport systems and synthesis of polyamines. PotF is a periplasmic binding protein of the polyamine transport system in *E. coli* [49]. It binds polyamines and transports them to the PotFGHI transport system. YdcS is an integral component of the YdcSTUV polyamine transport system [50]. Only the abundance of PotF and YdcS changed, while PotA amounts remained unchanged. PotA is also a component of a transport complex (PotABCD) with a structure similar to that of PotFGHI and YdcSTUV. *In* *silico* docking studies did not reveal a potential cause for the differential effects on structurally similar binding sites (Fig. S3). It is possible that the reduced abundance is not due to a structural change leading to reduced recognition, caused by binding of the sundew compounds to the transport protein, but rather to a metabolic effect. In contrast, SpeF is the enzyme ornithine decarboxylase, which catalyzes the decarboxylation of ornithine to putrescine. *E. coli* possesses two forms of ornithine decarboxylase: a constitutive form SpeC and an inducible form SpeF. While SpeC is present in all *E. coli* strains, SpeF is found only in some strains. The inducible ornithine decarboxylase is activated by low pH and the presence of ornithine in the growth medium [51]. In addition to ornithine, putrescine can also be synthesized from arginine. The study revealed a reduced abundance of the protein ArgT, which is part of the lysine/arginine/ornithine (LAO) transporter family responsible for transporting arginine from the periplasm into the cell. However, a pronounced change in the concentration of metabolites belonging to the arginine/ornithine metabolism could not be observed (Table S3 and S4).

Nevertheless, results suggest that the intracellular polyamine balance could be affected by treatment with *D*. *rotundifolia* L., potentially through disrupted polyamine uptake. This could have led to an intracellular spermidine deficiency, inhibiting biofilm formation. To further strengthen this hypothesis, the intracellular concentrations of spermidine and putrescine were analyzed by LC-MS/MS. The results show that the spermidine concentration was lower under the influence of *D*. *rotundifolia* L. compared to the growth control (Fig. 5c). Additionally, reduced arginine uptake might have decreased putrescine formation via the SpeA/SpeE synthesis pathway, possibly contributing to an increased induction of the alternative synthesis pathway involving SpeF. The ornithine decarboxylase SpeF catalyzes the formation of putrescine from ornithine and thus explains the elevated putrescine levels measured by LC-MS/MS. The above-mentioned increased concentrations of the metabolite citrate, which were observed during the metabolome analysis, could also indicate an increased degradation of putrescine to citrate (Fig. S2). Alternatively, the increase in citrate could indicate a blockage or weakening of the tricarboxylic acid (TCA) cycle, as a result of which citrate is no longer utilized. This could lead to a reduced energy supply by NADH and ATP, which could affect the overall energy metabolism of *E. coli* and thus negatively influence growth and biofilm formation.

The results of the study suggest that the flavonoids in the *D. rotundifolia* L. preparations could induce iron deficiency by complexing iron from the environment, which in turn influences biofilm formation in *E. coli*. A good indication of this is the complexation behavior of iron shown in the CAS assay, especially by 2″-*O*-galloylhyperoside. In addition, flavonoids and naphthoquinones could possibly reduce the intracellular polyamine concentration by influencing transporters.

However, it is difficult to precisely delineate the mechanism of action of naphthoquinones and flavonoids, as both groups of active substances could address several molecular target structures. For a comprehensive understanding of the mechanism of action of sundew, detailed molecular studies are necessary. Future research should focus on binding studies, knockout mutants, and the investigation of transport mechanisms.

## 3 Conclusion

The present study focuses on the plant *D. rotundifolia* L., which is cultivated in peatlands. This study investigates the bioactive compounds of *D. rotundifolia* L., especially flavonoids and naphthoquinones, which possess biofilm-inhibiting properties against multidrug-resistant, ESBL-producing *E. coli* strains, offering new therapeutic options. However, demands of modern phytomedicine are not only sustainable procurement of raw material, but also proof of safety and the provision of a mechanism of action. The results of the proteomic analysis support the hypothesis that similar signaling pathways are involved in biofilm inhibition for the main compounds of *D. rotundifolia* L. This is especially evident in the biofilm assay, where the same phenotype was observed across all treatment types: incomplete formation of the curli and cellulose matrix, resulting in biofilm discoloration. This consistency in phenotype suggests the potential to explore the underlying mechanisms in greater detail. It is challenging to determine whether the altered proteins are the cause of the changed phenotype or merely an expression of it. However, upon closer examination, it becomes evident that the single compound 2″-*O*-galloylhyperoside affects both iron and polyamine metabolism. This is also the case for the flavonoid fraction as well as in the crude extract, where 2″-*O*-galloylhyperoside is also present. The influence on iron metabolism might occur through the chelation of extracellular iron, a phenomenon phenotypically confirmed for flavonoids by a CAS assay. In contrast, this was not observed with 7-methyljuglone, where an influence on biofilm formation via the master regulator OmpR is conceivable. Overall, the data suggest that the two main groups of compounds in sundew, flavonoids and naphthoquinones, appear to target different pathways in biofilm formation and show a synergistic or additive effect. Contrary to previous assumptions that naphthoquinones are the primary active compounds, flavonoids also play a crucial role in the efficacy of *D*. *rotundifolia* L. [52,53]. Furthermore, toxicity data indicate that despite the presence of potentially cytotoxic naphthoquinones, the extract can be classified as safe. A combination of both compound groups as present in the extract, is therefore advantageous.

## 4 Experimental section

### 4.1 Material

Water, acetonitrile, dichloromethane (LC-MS grade, VWR, USA); hexadecyltrimethylammonium bromide, anhydrous piperazine, 5-sulfosalicylic acid, chrome azurol S, Triton X-100™, trypan blue, thiazolyl blue tetrazolium bromide (MTT), Coomassie Brilliant Blue G 250, ammonium bicarbonate (Sigma Aldrich, GER), sucrose, Na-EDTA (ethylenediaminetetraacetic acid disodium salt dihydrate), Tris-HCl (*tris*[hydroxymethyl]aminomethane hydrochloride), lysozyme, acid anhydrous acetic acid supra quality, tartaric acid, LB-medium Lennox, Nanoquant stock solution, dimethyl sulfoxide (DMSO), β-mercaptoethanol, ammonium sulfate, *o*-phosphoric acid, acetic acid, glycerol, sodium dodecyl sulfate (SDS) (Carl Roth GmbH, GER), bovine serum albumin, glycerol anhydrous for synthesis (Merck KGaA, GER), phosphate-buffered saline (PBS) with and without Ca^2+^/Mg^2+^, glutamine, DMEM/F10, gentamicin, penicillin/streptomycin/amphotericin B mix (PAN-Biotech GmbH, GER), EDTA (Biochrom AG, GER), DermaLife® basal medium (CellSystems GmbH, GER), human keratinocyte growth supplement (Life Technologies, GER), bromophenol blue (Oxford GlycoSystems, UK)

### 4.2 Bacterial strain

For this study, the CTX-*M*-15-ESBL-producing *E. coli* strain B2-ST131-O25, isolated from a dog with a urinary tract infection, was investigated (Data number: PBIO729, IMT17433; Genome accession numbers ERR163891). Despite its animal origin, this strain exhibits a close phylogenetic relationship with human strains [54]. It has been extensively studied in several investigations for its biofilm-forming properties and other virulence factors [[55], [56], [57]]. Bioinformatic analyses have identified virulence genes such as blaTEM-1, blaOXA-1, tet(A), tet(R), aadA, and aac (6’)-ib-cr, tet (A), strA, strB, aacC4, aac (6′)-IB-cr, blaTEM-1, as well as toxin-antitoxin system genes hok/sok, srnB/C, vagC/D, and pemI/K located on the ESBL (Extended Spectrum Beta-Lactamase) plasmid (CTX-*M*-15) [58]. Due to its broad spectrum of virulence genes, this strain can be considered a representative of the "ExPEC-ST131″ type (Extraintestinal Pathogenic *E. coli* Sequence Type 131). The *E. coli* cells were stored in a cryovial with a 20% glycerol solution at −80 °C. Planktonic cells were cultured by transferring a colony incubated for 24 h at 37 °C on LB agar (Luria/Miller) into 5 mL of LB medium and incubating overnight at 37 °C with shaking.

### 4.3 Plant material

Round-leaved sundew (*D*. *rotundifolia* L.), cultivated on *Sphagnum* spp. lawns in the Hankhauser Moor in northern Germany (53.26410 N, 8.27239 E), was obtained from Paludimed GmbH (GER). The aerial and subterranean parts of the plants were stored at −20 °C immediately after harvesting in July 2021. Prior to use, the plant material was dried in an oven at 50 °C for 24 h, subsequently pulverized, and sieved through a 500 μm mesh sieve.

### 4.4 Ultrasonic extraction

300 mg of the powdered *D*. *rotundifolia* L. biomass was mixed with 10 mL ethanol and sonicated at 50 °C for 15 min. Then, the sample was centrifuged at 3500 rpm for 5 min, the clear supernatant was decanted and 10 mL ethanol was added again to the residue. These steps were repeated three times in total and the supernatants were combined. The extract was concentrated under reduced pressure at 40 °C. The last traces of solvent were removed by evaporation at room temperature.

### 4.5 SPE-fractionation

To selectively enrich flavonoids and naphthoquinones, solid-phase extraction was performed according to the method of Gerschler et al. [3]. To obtain the flavonoid and naphthoquinone fractions, eluates with 42% and 100% methanol were collected and subsequently concentrated according to the method. The assignment of compound groups to the fractions is based on previous studies in which the constituents of the individual elution steps were quantified by HPLC (High Performance Liquid Chromatography) analysis [3]. The flavonoid fraction contained about 50% flavonoids (hyperoside, isoquercitrin, 2″-*O*-galloylhyperoside and quercetin), and the measured naphthoquinones 7-methyljuglone and plumbagin accounted for about 4% of the naphthoquinone fraction. In addition, this fraction contained about 1% quercetin, which could not be separated. The crude extract contained about 3% of the measured flavonoids and about 0.02% plumbagin and 7-methyljuglone.

### 4.6 Isolation of compounds

The methods for isolating single compounds are described in detail in the publication by Gerschler et al. [3] To obtain 2‘‘-*O*-galloylhyperoside, the dried flavonoid fraction was dissolved in 0.5 mL methanol and subjected to elution through a glass column (55 cm length, 1 cm diameter) filled with Sephadex® LH-20 (Amersham Biosciences, SWE), pre-swollen in methanol. One-milliliter fractions were collected and the area under the curve (AUC) of the peak with a retention time of 19.5 min was analyzed by HPLC. Fractions in which the AUC accounted for at least 90% of the chromatogram were combined, dried at room temperature, and stored at −20 °C.

For the isolation of 7-methyljuglone, 2 g of the pulverized plant material were mixed with 2 mL (0.33 g mL^−1^) of a tartaric acid and subsequently extracted with 150 mL *n*-hexane in a conventional Soxhlet apparatus. The extract was evaporated under reduced pressure and reconstituted in 800 μL dichloromethane. Isolation of 7-methyljuglone was achieved using a silica gel column, with methanol as the elution solvent. Individual bands were collected and analyzed by HPLC to identify the band corresponding to 7-methyljuglone.

### 4.7 Monolayer cell culture

The human squamous cell carcinoma cell line TR146 was cultured in DMEM/F10 supplemented with 2 mM glutamine and 10% fetal bovine serum at 37 °C in a humidified atmosphere (5% CO_2_). At a confluence of ∼80% cells were subcultured after checking the morphology. In brief, cells were washed with phosphate-buffered saline (PBS) without Ca^2+^/Mg^2+^ and then detached with 0.05% trypsin/0.02 mM EDTA for 10 min. Cell viability was measured by trypan blue exclusion. Cells were free of mycoplasma.

### 4.8 3D mucosa cell culture

Differentiation factors in the medium and the direct contact of the apical cell surface with air, while the basal surface is submerged with media, lead to multicellular growth of TR146 over time. This culturing method was carried out based on the method described by Zwicker et al. [59] with small modifications. ThinCert inserts from GBO with a membrane area of 0.336 cm^2^ and 0.4 μm pore diameter (Greiner Bio-One International GmbH, AUT) were placed into 24-well plates. 300 μL of a TR146 cell suspension (1 × 10^6^ cells mL^−1^) in media A was added to each insert. Medium A consisted of DermaLife® basal medium supplemented with 1% human keratinocyte growth supplement, 0.01 mg mL^−1^ gentamicin and 1% penicillin/streptomycin/amphotericin B mix. The basolateral side was provided with 600 μL medium A. After 24 h, the medium was removed and new was added to the different compartments (400 μL in the inserts; and 750 μL in the wells). After a further 24 h of incubation the medium was renewed again. The next day, the medium was removed completely, and the inserts were exposed to the air for 10 min in the laminar air flow box. The inserts were placed back into the 24-well plate and incubated with 750 μL medium B (medium A + 0.9 mM CaCl_2_). Every second day, the medium was replaced. On day 14, the transepithelial electrical resistance (TEER) was measured, and the mucosa equivalents were treated.

#### 4.8.1 Transepithelial electrical resistance measurement (TEER)

The inserts were washed twice with pre-warmed PBS with Ca^2+^/Mg^2+^. Afterwards, 400 μL PBS was filled into the inserts and 750 μL PBS was added to the wells. The TEER was measured to ensure the integrity of the mucosa equivalents using the EVOM™ Epithelial Volt-ohmmeter with the STX2-PLUS electrode (World Precision Instruments Ltd., GER). TEER was measured before (t:0 h) and after treatment (t:24 h). An insert without cells served as a blank.

#### 4.8.2 Treatment of 3D mucosa equivalents

Following the TEER measurement, inserts were placed into wells filled with 750 μL medium B. On top of the mucosa equivalents, 25 μL of extract solution prepared in PBS with Ca^2+/^Mg^2+^ was added and incubated for 24 h.

#### 4.8.3 Cell viability testing

Cell viability of the 3D mucosa equivalents after treatment was analyzed by using thiazolyl blue tetrazolium bromide (MTT). 600 μL of MTT-medium (0.5 mg mL^−1^) were added to the wells. Cells were further incubated for 3 h at 37 °C, following lysis with 500 μL solubilising buffer (0.04 M HCL in 2-propanol) for 18 h at 4 °C. The absorbance of the formazan solution was measured at 570 nm.

### 4.9 Determination of acute toxicity using *Galleria mellonella* larvae

*G. mellonella* larvae were purchased from proinsects GmbH (GER). Larvae were divided into groups each containing 10 individuals and weighed in petri dishes. 10 μL of *D*. *rotundifolia* L. extract solutions (crude extract, flavonoids and naphthoquinones fractions) were injected into the left proleg using a micro-syringe (Hamilton, GER). Untreated (survival) and solvent (20% DMSO) treated larvae were also implemented to exclude the possibility that death was caused by injection trauma. The larvae were then incubated at 37 °C and mortality was recorded every 24 h for four days. If the larvae showed no mobility and did not respond to physical stimuli, they were considered dead.

### 4.10 Macrocolony biofilm assay

To investigate the impact of *D*. *rotundifolia* L. on the protein abundance of *E. coli* biofilm, 150 μg mL^−1^ each of the extract, the naphthoquinone fraction, and 2‘‘-*O*-galloylhyperoside, as well as 100 μg mL^−1^ of the flavonoid fraction and 7-methyljuglone, were added to the wells of a 24-well plate. The amounts used were selected based on a previous study that demonstrated significant inhibition of biofilm production [3]. Methanol-containing stock solutions were prepared for this purpose. After solvent evaporation, 1 mL of Span-agar (consisting of 20 g LB-medium Lennox and 18 g Span-agar in 1 L DI water) was pipetted into each well. After the agar solidified, 5 μL of a bacterial suspension with an optical density of 0.5 was pipetted, briefly air-dried to remove excess water, and the plate was sealed with Parafilm®. The plate was incubated at 28 °C for 48 h.

### 4.11 Cell lysis

For cell lysis, macrocolonies were collected from Span-agar using an inoculation loop and suspended in a 1.5 mL screw-cap tube, pre-filled with 800 μL TE buffer (50 mM Tris-HCl, 10 mM EDTA, pH 7.5), and acid-washed glass beads (Part No. G4649-100G, Sigma-Aldrich, USA) with a diameter of less than 106 μm. The cell lysis was performed using a FastPrep-24™ benchtop homogenizer from MP Biomedicals™ (GER). The sample was subjected to four cycles of 1 min each at an acceleration of 6.5 m s^−2^, with the sample being placed on ice for 1 min between each cycle. Subsequently, the sample was centrifuged at 5000 *g* for 5 min at 4 °C. The supernatant was transferred to a new reaction tube and centrifuged again at 20,000 *g* for 20 min at 4 °C. The resulting supernatant was transferred to another reaction tube and stored at −20 °C.

### 4.12 Protein determination by bradford assay

For protein quantification, a calibration series ranging from 1.00 to 50.0 μg was prepared using stock solutions of bovine serum albumin dissolved in DI water (10 mg mL^−1^, 1 mg mL^−1^, and 0.1 mg mL^−1^). 20 μL of the sample were diluted 1:10 with DI water. Subsequently, 200 μL of the diluted solution and various concentrations of the calibration series were mixed with 800 μL of Nanoquant stock solution, previously diluted 1:5 with DI water. The mixtures were incubated for a period of 30 min at room temperature, protected from light. Absorbance was measured at 590 nm, and emission was recorded at 450 nm, using DI water as a blank.

### 4.13 Proteomic analysis

#### 4.13.1 Sample preparation

For the analysis, the respective sample volume equivalent to 20 μg of protein was mixed with 10 μL Laemmli sample buffer (10 mM Tris-HCl, 20% glycerol (w/v), 4% (w/v) sodium dodecyl sulfate (SDS), a trace amount of bromophenol blue, 3.75% (v/v) β-mercaptoethanol). The mixture was incubated at 98 °C for 15 min. Subsequently, the entire sample volume and 4 μL of a pre-stained protein ladder (Page Ruler™, Thermo Scientific, GER) were loaded onto a prepared 4–20% polyacrylamide gel (Criterion™ TGXTM Precast Gels, BioRad, USA). Electrophoresis was conducted using a Mini-PROTEAN Tetra Vertical Electrophoresis Cell (BioRad, USA) chamber at 180 V. The gels were then incubated in a fixation solution composed of 40% ethanol, 10% acetic acid (100%), and 50% DI water for 30 min and stained overnight in a staining solution (0.05% Coomassie Brilliant Blue G 250 - 5% [w/v] stock solution, 10% ammonium sulfate, 1% *o*-phosphoric acid). The following day, the gels were destained by multiple washes with DI water. Subsequently, each gel lane was fractionated into 10 gel pieces, which were further cut into smaller pieces. Each sample was incubated with 700 μL of gel washing solution (30% acetonitrile, 200 mM ammonium bicarbonate, 70% DI water) for 1 h at 37 °C on a shaker (900 rpm). After removing the washing solution, the samples were incubated again for 15 min at 37 °C and 900 rpm. This step was repeated until the gel pieces were destained. The samples were then dried in a vacuum centrifuge at 30 °C, covered with a 1:10 diluted trypsin solution (approximately 90 μL), and digested overnight at 37 °C and 1000 rpm. To elute the peptides from the gel, 100 μL MS-grade water was added to each sample the next day, and the samples were sonicated for 15 min. The peptide-containing supernatants were transferred to a new tube and dried using a vacuum centrifuge. Finally, the peptides were resuspended in a 0.1% (v/v) acetic acid solution.

#### 4.13.2 Proteomic-analysis

For the LC-MS/MS analyses, obtained peptides were separated by reversed phase column chromatography using an EASY nLC II (Thermo Fisher Scientific, USA) with self-packed columns (OD 360 μm, ID 100 μm, length 20 cm) filled with 3 μm diameter C18 particles (Dr. Maisch, GER). Following loading/desalting in 0.1% acetic acid in water, the peptides were separated by applying a binary non-linear gradient from 1 to 99% acetonitrile in 0.1% acetic acid over 80 min. The LC was coupled online to a LTQ Orbitrap Velos Pro mass spectrometer (Thermo Fisher, GER) with a spray voltage of 2.6 kV. After a survey scan in the Orbitrap (r = 30,000), MS/MS data were recorded for the twenty most intensive precursor ions in the linear ion trap. Singly charged ions were not considered for tandem Mass Spectrometry (MS/MS) analysis. The lock mass option was enabled throughout all analyses.

#### 4.13.3 Mass spectrometry (MS) data analysis

After mass spectrometric measurement, a database search against the strain database was obtained from the ENA-server (PBIO729=IMT17433 = ERR163891=Sanger Cambridge Number 8016_2_57, downloaded October 10, 2022, 4885 entries) was performed using MaxQuant (version 2.1.3.0). Common laboratory contaminants and reversed sequences were included by MaxQuant. Search parameters were set as follows: Trypsin/P specific digestion with up to two missed cleavages, methionine oxidation, and N-terminal acetylation as variable modification, match between runs with default parameters enabled. The FDRs (false discovery rates) of protein and PSM (peptide spectrum match) levels were set to 0.01. Two identified unique peptides were required for protein identification. MaxLFQ values were calculated in MaxQuant with default settings as a proxy for protein abundance.

#### 4.13.4 Data availability

The MS proteomics data discussed in this publication have been deposited to the ProteomeXchange Consortium via the PRIDE partner repository with the dataset identifier PXD055987 (Reviewer account details: Username, reviewer_pxd055987@ebi.ac.uk; Password, pPZXofoPgDrE).

### 4.14 Metabolome analysis

#### 4.14.1 Sample preparation

Sampling of macrocolony biofilms and metabolite extraction was done as described previously with minor modifications [60]. Colonies were scraped carefully from the surface of the agar plate and collected in an Eppendorf tube and the weight of the sample was determined. Four colonies were pooled after cultivation for 24 h and two colonies were pooled after cultivation for 48 h. Internal standard compounds for Liquid Chromatography–Mass Spectrometry (LC-MS) and Gas Chromatography–Mass Spectrometry (GC-MS) analysis, respectively, were added before metabolite extraction as described before [60,61]. For GC-MS analysis of intracellular metabolites, lyophilized samples were derivatized as described by Dörries et al. [62].

#### 4.14.2 Gas chromatography–mass spectrometry (GC/MS)-Analysis

Samples were analyzed with an Agilent 7890B GC system with an autosampler, an injector (G4513A), and a coupled mass selective detector (5977B MSD; Agilent, USA) by scan acquisition [60]. GC-MS parameters were used as follows: the injection volume of 1 μL was split 1:10, mass spectra were acquired within a mass range of 50–500 atomic mass units. All other parameters of GC and MSD were set as described before [62].

#### 4.14.3 GC/MS data-analysis

Identification of metabolites was carried out by comparison of mass spectra of peaks to mass spectra of pure standard compounds available from an in-house database using Agilent MassHunter Qualitative Analysis B.08.00 (Agilent, USA). Additional compounds were identified by library search using the database NIST 17. Metabolite identification was verified by matching the retention times and fragmentation patterns of detected peaks of a representative sample to those of analytical standard compounds measured within the same batch.

The quantification of metabolites was performed by normalizing areas of peaks to the area of peaks of internal standard compounds (relative amount = area metabolite/area internal standard) using Agilent MassHunter Quantitative Analysis B.08.00 as described in Leonard et al. [63] Relative amounts of metabolites were related to the wet weight of colonies.

#### 4.14.4 Liquid Chromatography–Mass Spectrometry (LC-MS/MS)-analysis

LC-MS/MS analysis was performed on an Agilent HPLC system (1200 series), coupled to an Agilent 6460 Triple quadrupole mass spectrometer with electrospray ionization source using a dynamic multiple reaction monitoring method in negative mode. The injection volume was 5 μL. LC- and MS-parameters were used as described by Rockstroh et al. [60].

#### 4.14.5 LC-MS/MS data-analysis

Metabolite concentrations were determined as described by Rockstroh et al. and related to the wet weight of colonies [60]. Agilent MassHunter Qualitative Analysis software and Agilent MassHunter Quantitative Analysis software (both version B.08.00) were used for MS data analysis.

#### 4.14.6 Data availability

Metabolom data that support the findings of this study are available from the corresponding author upon reasonable request.

### 4.15 CAS-assay

This assay was conducted based on the method described by Schwyn and Neilands (1987) [64]. First, 6 mL of a 10 mM hexadecyltrimethylammonium bromide (CTAB) stock solution was mixed with 40 mL of DI water. Separately, 1.5 mL of a FeCl_3_–HCl stock solution (1 mM FeCl_3_ in 10 mM HCl) was combined with 7.5 mL of a 2 mM Chrome Azurol S (CAS) dye solution. The Fe-CAS mixture was then added to the CTAB solution under continuous stirring. Next, 6.5 mL of 12 N HCl was slowly added to 25 mL of DI water, and 4.3 g of anhydrous piperazine was completely dissolved in this acid solution. This piperazine acid solution was then slowly mixed with the Fe-CAS-CTAB solution and brought to a final volume of 100 mL. Finally, sufficient 5-sulfosalicylic acid was added to achieve a final concentration of 4 mM. To evaluate the chelating agent properties of the *D*. *rotundifolia* L. extracts, a serial dilution of a 1 mg mL^−1^ stock solution of extract, the fractions, 7-methyljuglone and 2″-*O*-galloylhyperoside with ethanol was prepared to 100 μL of each concentration, 100 μL of the CAS solution was added resulting in a final concentration ranging from 200 to 6.25 μg mL^−1^. After incubating for 1 h at room temperature in the dark, the absorption was measured at 630 nm. To account for the intrinsic absorption of the samples, each dilution series without the CAS dye was measured, and these values were subtracted from the measurements with the CAS dye. As a control, an ethanol-water mixture with 100 μL CAS solution was used. The results were evaluated by calculating the ratio of the absorption of the control to the absorption of the samples. A ratio of 1 indicates the absence of chelators in the sample. The assay was performed in three technical as well as three biological replicates.

### 4.16 Quantification of the intracellular spermidine and putrescine concentration

To analyze the intracellular concentrations of spermidine and putrescine, the macrocolonies generated with the macrocolony assay were transferred into a 1.5-mL reaction vessel using an inoculation loop. Cell extraction was performed through a heat lysis method. Initially, the macrocolony was suspended in 200 μL of STET-buffer (8% sucrose, 5% Triton X-100™, 50 mM Sodium-EDTA, 50 mM Tris-HCl, pH 8.0), followed by enrichment with 15 μL of a lysis buffer (10 mg lysozyme per 1.5 mL TE-buffer [10 mM Tris-HCl, 1 mM NA-EDTA, pH 8.0]). Following this preparation, the cell suspension was heated for 1 min at 90 °C Celsius and then centrifuged (15 min, 20,800 g). The supernatant was transferred to a new reaction vessel and stored at −20 °C. Concentration analysis was conducted according to the methodology of Senekowitsch et al. [65]. The calibration range was adjusted to 60–15000 ng mL-1 for both polyamines, the concentrations of the internal standards were 10 μg mL-1, for spermidine 2 μL were injected, while for putrescine it was 0.5 μL.

### 4.17 Molecular modeling

The open apo states structures of PotD and YdcS were predicted using targeted molecular dynamics simulations based on the available crystal structures of PotF. The closed state structures of PotD (PDB: 1POT), YdcS (PDB: 6NLP), PotF (PDB: 6YE0), and the open apo state structure of PotF (PDB: 6YED) were first aligned to the homologous region, which does not undergo any conformational changes between open and closed state. All structures were then prepared using the Protein Preparation Wizard [66] in Maestro (Schrödinger Release 2024-2). This included adding hydrogen atoms, replacing partially missing side chain atoms, optimizing the protonation states, and minimizing the final constraint minimization with the OPLS4 force field [67]. The simulation systems were generated with CHARMM-GUI using the CHARMM36 m [68] force field for NAMD 2.14 (GPU version) [69] for both 1POT and 6NLP. The water model was TIP3P, and hydrogen mass repartitioning was applied. For the TMD simulations, a protocol consisting of a minimization for 10,000 steps, followed by a short constraint equilibration (*NPT*) for 1 ns and the actual pulling phase (*NPT*) for 10 ns was used. The temperature and pressure were maintained by a Langevin thermostat and a Langevin barostat, respectively. The time step was set to 4 fs and a snapshot was written every 20 ps. The actual pulling was performed with the colvars module [70] and a linearly increasing force constant. The initial force constant was 1 kJ/mol/U^2^ and increased to the target value of 25 kJ/mol/U^2^ in the course of the simulation. For the reference points, all secondary structure elements were taken into account that are identical in all structures and have only undergone positional changes from closed to open state in PotF. The final structures were minimized for a further 20,000 steps. Molecular docking of the investigated compounds was carried out with the obtained structures using SiteMap [71] and Glide SP [72] (Schrödinger Release 2024-2), but no clear binding poses could be obtained after visual evaluation.

The structures of the complexes PotABCD, PotFGHI and YdcSTUV were generated using AlphaFold3 (AlphaFold Server Beta) [73]. Two entities as well as two molecules of ATP were used for the respective proteins PotA, PotG and YdcT.3.

## CRediT authorship contribution statement

**Sandy Gerschler:** Writing – review & editing, Writing – original draft, Visualization, Investigation, Formal analysis, Data curation. **Sandra Maaß:** Writing – original draft, Formal analysis, Data curation, Conceptualization. **Philip Gerth:** Writing – review & editing, Investigation, Formal analysis, Data curation. **Lukas Schulig:** Writing – review & editing, Writing – original draft, Formal analysis, Data curation. **Toni Wildgrube:** Writing – review & editing, Writing – original draft, Investigation, Data curation. **Jan Rockstroh:** Investigation, Formal analysis, Data curation. **Martina Wurster:** Writing – review & editing, Investigation, Formal analysis, Data curation, Conceptualization. **Karen Methling:** Writing – review & editing, Writing – original draft, Investigation, Formal analysis, Data curation, Conceptualization. **Dörte Becher:** Supervision, Conceptualization. **Michael Lalk:** Supervision, Conceptualization. **Christian Schulze:** Writing – review & editing, Conceptualization. **Sebastian Guenther:** Writing – review & editing, Supervision, Project administration, Funding acquisition, Conceptualization. **Nadin Schultze:** Writing – review & editing, Writing – original draft, Visualization, Methodology, Formal analysis, Data curation, Conceptualization.

## Conflict of interest

The authors declare no conflicts of interest.

## Data Availability

Data will be made available on request.
