# Supplementary material for: Drosera rotundifolia L. as E. coli biofilm inhibitor: Insights into the mechanism of action using proteomics/metabolomics and toxicity studies
Source: Biofilm. 2025 Feb 28;9:100268. doi: 10.1016/j.bioflm.2025.100268 (PMC11930149; doi:10.1016/j.bioflm.2025.100268)
Supplement: Multimedia component 1 [file mmc1.docx]

**Supporting information**

**Table S1.** Changes of metabolites measured by LC-MS in biofilm colonies treated for 24h with extracts, fractions and compounds. Mean of relative amount/wet weight biofilm of three independent biological replicates. Log2fc >0.8; Log2FC<-0.8. two-way ANOVA, post-hoc Dunnett Test; *p<0.0332; **p<0.0021; ***p<0.0002; ****p<0.0001

|  | **24 h** | | | | | | | | | | | | | | | | | | |
| --- | --- | --- | --- | --- | --- | --- | --- | --- | --- | --- | --- | --- | --- | --- | --- | --- | --- | --- | --- |
|  | **EtOH** | | **D. rotundifolia** | | | **42 % Frac** | | | **100% Frac** | | | **EtOH** | | **7-MJ** | | | **GH** | | |
|  | **Mean** | **SD** | **Mean** | **SD** | **Log2 fc** | **Mean** | **SD** | **Log2 fc** | **Mean** | **SD** | **Log2 fc** | **Mean** | **MW** | **Mean** | **SD** | **Log2 fc** | **Mean** | **SD** | **Log2 fc** |
| **Ribose-5-phosphate** | 0.0286 | 0.0171 | 0.0072 | 0.0059 | -2.0 | 0.0088 | 0.0062 | -1.7 | 0.0075 | 0.0058 | -1.9 | 0.0285 | 0.0086 | 0.0146 | 0.0085 | -1.0 | 0.0077 | 0.0067 | -1.9 |
| **CMP** | 0.0103 | 0.0010 | 0.0034 | 0.0010 | -1.6 | 0.0077 | 0.0018 | -0.4 | 0.0036 | 0.0011 | -1.5 | 0.0122 | 0.0032 | 0.0086 | 0.0034 | -0.5 | 0.0093 | 0.0020 | -0.4 |
| **UMP** | 0.0628 | 0.0131  * | 0.0284 | 0.0127 | -1.1 | 0.0406 | 0.0200 | -0.6 | 0.0240  ** | 0.0062 | -1.4 | 0.0773 | 0.0208 | 0.0563 | 0.0125 | -0.5 | 0.0444  * | 0.0148 | -0.8 |
| IMP | 0.0228 | 0.0045 | 0.0158 | 0.0050 | -0.5 | 0.0236 | 0.0053 | 0.1 | 0.0160 | 0.0049 | -0.5 | 0.0176 | 0.0077 | 0.0168 | 0.0038 | -0.1 | 0.0240 | 0.0031 | 0.4 |
| GMP | 0.0006 | 0.0001 | 0.0004 | 0.0001 | -0.6 | 0.0006 | 0.0001 | -0.1 | 0.0004 | 0.0001 | -0.5 | 0.0006 | 0.0001 | 0.0005 | 0.0001 | -0.1 | 0.0007 | 0.0001 | 0.1 |
| AMP | 0.0318 | 0.0100 | 0.0234 | 0.0091 | -0.4 | 0.0237 | 0.0061 | -0.4 | 0.0221 | 0.0072 | -0.5 | 0.0300 | 0.0018 | 0.0246 | 0.0049 | -0.3 | 0.0215 | 0.0043 | -0.5 |
| UDP-Glc | 0.1474 | 0.0662 | 0.1742 | 0.0465 | 0.2 | 0.2015  **** | 0.0692 | 0.5 | 0.2186  **** | 0.0496 | 0.6 | 0.1691 | 0.0796 | 0.1720 | 0.0324 | 0.0 | 0.1625 | 0.0704 | -0.1 |
| **UDP-GlcNAc** | 0.0134 | 0.0038 | 0.0161 | 0.0023 | 0.3 | 0.0241 | 0.0076 | 0.8 | 0.0253 | 0.0012 | 0.9 | 0.0132 | 0.0039 | 0.0167 | 0.0030 | 0.3 | 0.0200 | 0.0060 | 0.6 |
| CDP | 0.0087 | 0.0008 | 0.0090 | 0.0015 | 0.0 | 0.0094 | 0.0011 | 0.1 | 0.0085 | 0.0013 | 0.0 | 0.0094 | 0.0013 | 0.0084 | 0.0019 | -0.2 | 0.0089 | 0.0009 | -0.1 |
| 6-Phosphogluconate | 0.0052 | 0.0011 | 0.0043 | 0.0014 | -0.3 | 0.0043 | 0.0005 | -0.3 | 0.0043 | 0.0009 | -0.3 | 0.0067 | 0.0011 | 0.0057 | 0.0009 | -0.2 | 0.0043 | 0.0008 | -0.6 |
| GDP | 0.0044 | 0.0004 | 0.0051 | 0.0003 | 0.2 | 0.0052 | 0.0004 | 0.2 | 0.0048 | 0.0005 | 0.1 | 0.0043 | 0.0007 | 0.0039 | 0.0006 | -0.2 | 0.0044 | 0.0005 | 0.0 |
| UDP | 0.0056 | 0.0008 | 0.0058 | 0.0011 | 0.1 | 0.0064 | 0.0012 | 0.2 | 0.0062 | 0.0005 | 0.2 | 0.0063 | 0.0015 | 0.0061 | 0.0016 | 0.0 | 0.0059 | 0.0013 | -0.1 |
| Frc 1,6-bP | 0.0057 | 0.0010 | 0.0054 | 0.0021 | -0.1 | 0.0058 | 0.0006 | 0.0 | 0.0067 | 0.0001 | 0.2 | 0.0062 | 0.0020 | 0.0047 | 0.0016 | -0.4 | 0.0061 | 0.0010 | 0.0 |
| ADP | 0.0263 | 0.0029 | 0.0181 | 0.0023 | -0.5 | 0.0199 | 0.0021 | -0.4 | 0.0175 | 0.0023 | -0.6 | 0.0267 | 0.0024 | 0.0230 | 0.0045 | -0.2 | 0.0198 | 0.0042 | -0.4 |
| CTP | 0.0107 | 0.0003 | 0.0107 | 0.0010 | 0.0 | 0.0109 | 0.0016 | 0.0 | 0.0107 | 0.0008 | 0.0 | 0.0116 | 0.0018 | 0.0098 | 0.0021 | -0.2 | 0.0105 | 0.0010 | -0.1 |
| UDP-GlcA | 0.0080 | 0.0028 | 0.0065 | 0.0023 | -0.3 | 0.0099 | 0.0042 | 0.3 | 0.0095 | 0.0049 | 0.3 | 0.0101 | 0.0027 | 0.0111 | 0.0024 | 0.1 | 0.0096 | 0.0050 | -0.1 |
| pRpp | 0.0034 | 0.0014 | 0.0044 | 0.0015 | 0.4 | 0.0037 | 0.0011 | 0.1 | 0.0044 | 0.0023 | 0.4 | 0.0036 | 0.0012 | 0.0028 | 0.0013 | -0.3 | 0.0028 | 0.0012 | -0.4 |
| GTP | 0.0044 | 0.0003 | 0.0050 | 0.0001 | 0.2 | 0.0050 | 0.0002 | 0.2 | 0.0046 | 0.0006 | 0.1 | 0.0043 | 0.0008 | 0.0039 | 0.0008 | -0.1 | 0.0047 | 0.0011 | 0.1 |
| UTP | 0.0088 | 0.0014 | 0.0090 | 0.0018 | 0.0 | 0.0099 | 0.0018 | 0.2 | 0.0093 | 0.0007 | 0.1 | 0.0101 | 0.0026 | 0.0097 | 0.0026 | -0.1 | 0.0094 | 0.0021 | -0.1 |
| ATP | 0.0268 | 0.0023 | 0.0188 | 0.0019 | -0.5 | 0.0212 | 0.0023 | -0.3 | 0.0184 | 0.0024 | -0.5 | 0.0281 | 0.0031 | 0.0244 | 0.0047 | -0.2 | 0.0213 | 0.0043 | -0.4 |
| CoA | 0.0005 | 0.0002 | 0.0004 | 0.0002 | -0.1 | 0.0007 | 0.0003 | 0.6 | 0.0007 | 0.0005 | 0.7 | 0.0003 | 0.0001 | 0.0003 | 0.0001 | -0.2 | 0.0004 | 0.0001 | 0.4 |
| AcCoA | 0.0003 | 0.0001 | 0.0003 | 0.0001 | -0.4 | 0.0005 | 0.0003 | 0.7 | 0.0005 | 0.0002 | 0.6 | 0.0004 | 0.0002 | 0.0005 | 0.0001 | 0.2 | 0.0004 | 0.0002 | 0.0 |

**Table S2.** Changes of metabolites measured by LC-MS in biofilm colonies treated for 48h with extracts, fractions and compounds. Mean of relative amount/wet weight biofilm of four independent biological replicates. Log2fc >0.8; Log2FC<-0.8. two-way ANOVA, post-hoc Dunnett Test; *p<0.0332; **p<0.0021; ***p<0.0002; ****p<0.0001

|  | **48h** | | | | | | | | | | | | | | | | | | |
| --- | --- | --- | --- | --- | --- | --- | --- | --- | --- | --- | --- | --- | --- | --- | --- | --- | --- | --- | --- |
|  | **EtOH** | | **D. rotundifolia** | | | **42 % Frac** | | | **100% Frac** | | | **EtOH** | | **7-MJ** | | | **GH** | | |
|  | **Mean** | **SD** | **Mean** | **SD** | **Log2 fc** | **Mean** | **SD** | **Log2 fc** | **Mean** | **SD** | **Log2 fc** | **Mean** | **MW** | **Mean** | **SD** | **Log2 fc** | **Mean** | **SD** | **Log2 fc** |
| **Ribose-5-phosphate** | 0.0289 | 0.0244 | 0.0109 | 0.0083 | -1.4 | 0.0136 | 0.0069 | -1.1 | 0.0125 | 0.0037 | -1.2 | 0.0262 | 0.0092 | 0.0153 | 0.009 | -0.8 | 0.0104  ** | 0.0067 | -1.3 |
| CMP | 0.0088 | 0.0047 | 0.0101 | 0.0051 | 0.2 | 0.0065 | 0.0051 | -0.4 | 0.0083 | 0.0034 | -0.1 | 0.0068 | 0.0016 | 0.0086 | 0.0034 | 0.3 | 0.0092 | 0.0033 | 0.4 |
| **UMP** | 0.0393 | 0.014 | 0.0917  **** | 0.0808 | 1.2 | 0.0968  **** | 0.0918 | 1.3 | 0.0864  *** | 0.0705 | 1.1 | 0.0557 | 0.035 | 0.0498 | 0.0257 | -0.2 | 0.0656 | 0.0165 | 0.2 |
| **IMP** | 0.0096 | 0.0055 | 0.0047 | 0.0022 | -1.0 | 0.0054 | 0.0042 | -0.8 | 0.0051 | 0.0055 | -0.9 | 0.0094 | 0.0054 | 0.0103 | 0.0051 | 0.1 | 0.0082 | 0.0019 | -0.2 |
| GMP | 0.0004 | 8E-05 | 0.0006 | 0.0001 | 0.4 | 0.0005 | 0.0001 | 0.3 | 0.0007 | 0.0002 | 0.8 | 0.0005 | 6E-05 | 0.0005 | 7E-05 | 0.1 | 0.0005 | 0.0002 | 0.2 |
| AMP | 0.0256 | 0.0065 | 0.0338 | 0.0123 | 0.4 | 0.0439 | 0.0076 | 0.8 | 0.0553  * | 0.023 | 1.1 | 0.0293 | 0.0061 | 0.0256 | 0.0042 | -0.2 | 0.0316 | 0.0088 | 0.1 |
| UDP-Glc | 0.101 | 0.0194 | 0.0808 | 0.0151 | -0.3 | 0.0835 | 0.0261 | -0.3 | 0.0703  * | 0.0228 | -0.5 | 0.0924 | 0.0174 | 0.1045  * | 0.0151 | 0.2 | 0.0933 | 0.016 | 0.0 |
| UDP-GlcNAc | 0.011 | 0.0013 | 0.0111 | 0.0015 | 0.0 | 0.0114 | 0.0042 | 0.0 | 0.0098 | 0.0039 | -0.2 | 0.01 | 0.0015 | 0.0117 | 0.0015 | 0.2 | 0.0122 | 0.0014 | 0.3 |
| CDP | 0.0094 | 0.0025 | 0.0087 | 0.0026 | -0.1 | 0.0066 | 0.003 | -0.5 | 0.0058 | 0.0028 | -0.7 | 0.0106 | 0.003 | 0.0113 | 0.0018 | 0.1 | 0.0089 | 0.0016 | -0.3 |
| **6-Phospho-gluconate** | 0.0097 | 0.0028 | 0.0045 | 0.0007 | -1.1 | 0.0046 | 0.002 | -1.1 | 0.003 | 0.0012 | -1.7 | 0.0087 | 0.0024 | 0.0112 | 0.0005 | 0.4 | 0.0077 | 0.0006 | -0.2 |
| GDP | 0.0037 | 0.0003 | 0.0037 | 0.0005 | 0.0 | 0.0036 | 0.0011 | 0.0 | 0.0033 | 0.0007 | -0.1 | 0.0033 | 0.0006 | 0.0036 | 0.0005 | 0.1 | 0.0036 | 0.0004 | 0.1 |
| UDP | 0.0044 | 0.0006 | 0.0047 | 0.0008 | 0.1 | 0.0047 | 0.0015 | 0.1 | 0.0044 | 0.001 | 0.0 | 0.004 | 0.0005 | 0.0043 | 0.0004 | 0.1 | 0.0046 | 0.0007 | 0.2 |
| **Frc 1,6-bP** | 0.0102 | 0.0101 | 0.0036 | 0.001 | -1.5 | 0.0038 | 0.0015 | -1.4 | 0.003 | 0.0009 | -1.8 | 0.0057 | 0.0025 | 0.0057 | 0.0018 | 0.0 | 0.005 | 0.0016 | -0.2 |
| ADP | 0.0219 | 0.0016 | 0.0262 | 0.0068 | 0.3 | 0.0227 | 0.0079 | 0.1 | 0.0219 | 0.0048 | 0.0 | 0.0228 | 0.0028 | 0.0228 | 0.0019 | 0.0 | 0.025 | 0.002 | 0.1 |
| CTP | 0.0115 | 0.0021 | 0.0109 | 0.0008 | -0.1 | 0.0097 | 0.0022 | -0.3 | 0.0087 | 0.0022 | -0.4 | 0.0122 | 0.0025 | 0.0125 | 0.0021 | 0.0 | 0.0111 | 0.0021 | -0.1 |
| UDP-GlcA | 0.0086 | 0.0008 | 0.0107 | 0.0024 | 0.3 | 0.0099 | 0.004 | 0.2 | 0.0093 | 0.0033 | 0.1 | 0.0095 | 0.0009 | 0.0094 | 0.001 | 0.0 | 0.0118 | 0.0014 | 0.3 |
| pRpp | 0.0021 | 0.0008 | 0.0017 | 0.0011 | -0.3 | 0.0015 | 0.0014 | -0.5 | 0.0015 | 0.0015 | -0.5 | 0.002 | 0.0008 | 0.0018 | 0.0008 | -0.2 | 0.0014 | 0.0003 | -0.5 |
| GTP | 0.0036 | 0.0005 | 0.0036 | 0.0006 | 0.0 | 0.0036 | 0.0011 | 0.0 | 0.0033 | 0.0007 | -0.2 | 0.0033 | 0.0005 | 0.0035 | 0.0003 | 0.1 | 0.0036 | 0.0005 | 0.1 |
| UTP | 0.0066 | 0.0006 | 0.0072 | 0.0015 | 0.1 | 0.0071 | 0.0024 | 0.1 | 0.0061 | 0.002 | -0.1 | 0.0061 | 0.0009 | 0.0069 | 0.0007 | 0.2 | 0.0073 | 0.001 | 0.3 |
| ATP | 0.0225 | 0.0008 | 0.027 | 0.0083 | 0.3 | 0.0232 | 0.0081 | 0.0 | 0.0206 | 0.0068 | -0.1 | 0.0239 | 0.0036 | 0.0239 | 0.0017 | 0.0 | 0.0256 | 0.0012 | 0.1 |
| **CoA** | 0.0003 | 0.0002 | 0.0003 | 0.0001 | 0.0 | 0.0004 | 0.0002 | 0.4 | 0.0005 | 0.0003 | 0.6 | 0.0004 | 0.0002 | 0.0002 | 7E-05 | -0.7 | 0.0003 | 0.0001 | -0.3 |
| **AcCoA** | 0.0008 | 0.0004 | 0.0007 | 0.0003 | -0.3 | 0.0007 | 0.0003 | -0.3 | 0.0007 | 0.0003 | -0.3 | 0.001 | 0.0004 | 0.0009 | 0.0002 | -0.2 | 0.0007 | 0.0002 | -0.6 |

**Table S3.** Changes of metabolites measured by GC-MS in biofilm colonies treated for 24h with extracts, fractions and compounds. Mean of relative amount/wet weight biofilm of three independent biological replicates. Log2fc >0.8; Log2FC<-0.8. two-way ANOVA, post-hoc Dunnett Test; ****p<0.0001

|  | **24h** | | | | | | | | | | | | | | | | | | |
| --- | --- | --- | --- | --- | --- | --- | --- | --- | --- | --- | --- | --- | --- | --- | --- | --- | --- | --- | --- |
|  | **EtOH** | | **D. rotundifolia** | | | **42 % Frac** | | | **100% Frac** | | | **EtOH** | | **7-MJ** | | | **GH** | | |
|  | **Mean** | **SD** | **Mean** | **SD** | **Log2 fc** | **Mean** | **SD** | **Log2 fc** | **Mean** | **SD** | **Log2 fc** | **Mean** | **MW** | **Mean** | **SD** | **Log2 fc** | **Mean** | **SD** | **Log2 fc** |
| pyruvate | 0.0120 | 0.0042 | 0.0114 | 0.0047 | -0.1 | 0.0107 | 0.0036 | -0.2 | 0.0118 | 0.0038 | 0.0 | 0.0115 | 0.0051 | 0.0105 | 0.0038 | -0.1 | 0.0104 | 0.0047 | -0.1 |
| **alanine** | 0.0160 | 0.0079 | 0.0286 | 0.0115 | 0.8 | 0.0221 | 0.0085 | 0.5 | 0.0250 | 0.0084 | 0.6 | 0.0143 | 0.0086 | 0.0127 | 0.0088 | -0.2 | 0.0180 | 0.0107 | 0.3 |
| 3-hydroxybutyrate | 4.73E-05 | 1.28E-05 | 4.49E-05 | 1.47E-05 | -0.1 | 4.32E-05 | 1.58E-05 | -0.1 | 4.20E-05 | 1.83E-05 | -0.2 | 5.74E-05 | 4.07E-05 | 4.88E-05 | 1.11E-05 | -0.2 | 3.91E-05 | 7.23E-06 | -0.6 |
| valine | 0.1251 | 0.0085 | 0.1304 | 0.0063 | 0.1 | 0.1313 | 0.0065 | 0.1 | 0.1359 | 0.0107 | 0.1 | 0.1272 | 0.0053 | 0.1260 | 0.0080 | 0.0 | 0.1352 | 0.0064 | 0.1 |
| urea | 0.0027 | 0.0007 | 0.0017 | 0.0002 | -0.7 | 0.0021 | 0.0001 | -0.4 | 0.0019 | 0.0004 | -0.5 | 0.0017 | 0.0004 | 0.0023 | 0.0004 | 0.4 | 0.0024 | 0.0002 | 0.5 |
| leucine | 0.1319 | 0.0137 | 0.1540 | 0.0153 | 0.2 | 0.1473 | 0.0169 | 0.2 | 0.1446 | 0.0106 | 0.1 | 0.1326 | 0.0268 | 0.1467 | 0.0153 | 0.1 | 0.1600 | 0.0168 | 0.3 |
| isoleucine | 0.1115 | 0.0029 | 0.1147 | 0.0074 | 0.0 | 0.1130 | 0.0067 | 0.0 | 0.1164 | 0.0069 | 0.1 | 0.1138 | 0.0065 | 0.1125 | 0.0073 | 0.0 | 0.1201 | 0.0082 | 0.1 |
| proline | 0.0068 | 0.0040 | 0.0070 | 0.0034 | 0.0 | 0.0055 | 0.0017 | -0.3 | 0.0054 | 0.0019 | -0.3 | 0.0060 | 0.0038 | 0.0058 | 0.0043 | -0.1 | 0.0056 | 0.0028 | -0.1 |
| glycine | 0.0058 | 0.0010 | 0.0060 | 0.0012 | 0.1 | 0.0054 | 0.0005 | -0.1 | 0.0068 | 0.0009 | 0.2 | 0.0055 | 0.0015 | 0.0054 | 0.0010 | 0.0 | 0.0062 | 0.0003 | 0.2 |
| succinate | 0.0260 | 0.0140 | 0.0230 | 0.0062 | -0.2 | 0.0228 | 0.0091 | -0.2 | 0.0219 | 0.0097 | -0.2 | 0.0208 | 0.0092 | 0.0182 | 0.0146 | -0.2 | 0.0205 | 0.0153 | 0.0 |
| fumarate | 0.0052 | 0.0044 | 0.0045 | 0.0035 | -0.2 | 0.0037 | 0.0025 | -0.5 | 0.0036 | 0.0025 | -0.5 | 0.0043 | 0.0038 | 0.0039 | 0.0036 | -0.1 | 0.0038 | 0.0032 | -0.2 |
| **serine** | 0.0070 | 0.0011 | 0.0067 | 0.0013 | -0.1 | 0.0064 | 0.0010 | -0.1 | 0.0091 | 0.0015 | 0.4 | 0.0055 | 0.0017 | 0.0060 | 0.0014 | 0.1 | 0.0140 | 0.0093 | 1.3 |
| threonine | 0.0040 | 0.0006 | 0.0045 | 0.0013 | 0.2 | 0.0044 | 0.0009 | 0.1 | 0.0056 | 0.0006 | 0.5 | 0.0038 | 0.0007 | 0.0041 | 0.0002 | 0.1 | 0.0059 | 0.0008 | 0.6 |
| **beta-alanine** | 0.0008 | 0.0003 | 0.0006 | 0.0001 | -0.4 | 0.0006 | 0.0001 | -0.6 | 0.0004 | 0.0002 | -1.0 | 0.0008 | 0.0004 | 0.0007 | 0.0004 | -0.2 | 0.0005 | 0.0002 | -0.6 |
| malate | 0.0077 | 0.0038 | 0.0090 | 0.0050 | 0.2 | 0.0081 | 0.0031 | 0.1 | 0.0083 | 0.0030 | 0.1 | 0.0076 | 0.0051 | 0.0074 | 0.0042 | 0.0 | 0.0074 | 0.0040 | 0.0 |
| methionine | 0.0630 | 0.0012 | 0.0621 | 0.0061 | 0.0 | 0.0633 | 0.0053 | 0.0 | 0.0676 | 0.0055 | 0.1 | 0.0647 | 0.0068 | 0.0633 | 0.0053 | 0.0 | 0.0647 | 0.0038 | 0.0 |
| 5-oxoproline | 0.1277 | 0.0118 | 0.1323 | 0.0114 | 0.1 | 0.1324 | 0.0119 | 0.1 | 0.1351 | 0.0168 | 0.1 | 0.1282 | 0.0099 | 0.1307 | 0.0166 | 0.0 | 0.1451 | 0.0235 | 0.2 |
| aspartate | 0.1065 | 0.0258 | 0.1125 | 0.0276 | 0.1 | 0.1065 | 0.0240 | 0.0 | 0.1136 | 0.0221 | 0.1 | 0.1023 | 0.0217 | 0.1147 | 0.0357 | 0.2 | 0.1013 | 0.0117 | 0.0 |
| **4-hydroxyroline** | 9.35E-05 | 6.82E-05 | 7.13E-05 | 4.02E-05 | -0.4 | 4.44E-05 | 1.32E-05 | -1.1 | 4.80E-05 | 9.07E-06 | -1.0 | 4.93E-05 | 1.14E-05 | 4.61E-05 | 1.99E-05 | -0.1 | 5.00E-05 | 9.54E-06 | 0.0 |
| cysteine | 0.0010 | 0.0007 | 0.0007 | 0.0005 | -0.5 | 0.0007 | 0.0004 | -0.5 | 0.0007 | 0.0005 | -0.5 | 0.0010 | 0.0008 | 0.0010 | 0.0008 | 0.1 | 0.0006 | 0.0004 | -0.7 |
| 2-oxoglutarat | 0.0012 | 0.0000 | 0.0013 | 0.0002 | 0.0 | 0.0015 | 0.0001 | 0.3 | 0.0014 | 0.0002 | 0.2 | 0.0013 | 0.0003 | 0.0013 | 0.0002 | 0.0 | 0.0014 | 0.0003 | 0.1 |
| phosphoenol-  pyruvate | 0.1132 | 0.0182 | 0.1085 | 0.0331 | -0.1 | 0.1143 | 0.0300 | 0.0 | 0.1144 | 0.0333 | 0.0 | 0.1042 | 0.0192 | 0.1094 | 0.0275 | 0.1 | 0.1095 | 0.0299 | 0.1 |
| glutamate | 0.1767 | 0.0024 | 0.1886 | 0.0093 | 0.1 | 0.1847 | 0.0088 | 0.1 | 0.1949 | 0.0112 | 0.1 | 0.1807 | 0.0080 | 0.1782 | 0.0089 | 0.0 | 0.1906 | 0.0107 | 0.1 |
| **phenylalanine** | 0.1699 | 0.2567 | 0.0513  **** | 0.0494 | -1.7 | 0.0173  **** | 0.0150 | -3.3 | 0.1815 | 0.2640 | 0.1 | 0.0434 | 0.0469 | 0.0130 | 0.0114 | -1.7 | 0.0305 | 0.0089 | -0.5 |
| dihydroxyaceton-phosphat | 0.0001 | 0.0000 | 0.0001 | 0.0000 | -0.1 | 0.0001 | 0.0000 | -0.2 | 0.0001 | 0.0000 | 0.0 | 0.0001 | 0.0001 | 0.0001 | 0.0000 | -0.4 | 0.0001 | 0.0000 | -0.2 |
| glutamine | 0.0027 | 0.0004 | 0.0029 | 0.0001 | 0.1 | 0.0030 | 0.0001 | 0.1 | 0.0033 | 0.0001 | 0.3 | 0.0025 | 0.0005 | 0.0028 | 0.0005 | 0.2 | 0.0028 | 0.0001 | 0.2 |
| 2-phospho-  glycerate | 0.0005 | 0.0001 | 0.0005 | 0.0001 | -0.2 | 0.0005 | 0.0001 | 0.1 | 0.0006 | 0.0001 | 0.2 | 0.0007 | 0.0000 | 0.0007 | 0.0001 | 0.0 | 0.0006 | 0.0001 | -0.1 |
| 3-phospho-  glycerate | 0.0077 | 0.0020 | 0.0065 | 0.0004 | -0.2 | 0.0079 | 0.0002 | 0.0 | 0.0086 | 0.0014 | 0.2 | 0.0083 | 0.0015 | 0.0088 | 0.0026 | 0.1 | 0.0077 | 0.0005 | -0.1 |
| ornithine | 0.0030 | 0.0006 | 0.0036 | 0.0003 | 0.3 | 0.0034 | 0.0003 | 0.2 | 0.0032 | 0.0005 | 0.1 | 0.0025 | 0.0004 | 0.0026 | 0.0004 | 0.1 | 0.0042 | 0.0009 | 0.8 |
| **citrate** | 0.0031 | 0.0006 | 0.0158 | 0.0046 | 2.4 | 0.0134 | 0.0057 | 2.1 | 0.0190 | 0.0065 | 2.6 | 0.0027 | 0.0007 | 0.0028 | 0.0009 | 0.1 | 0.0073 | 0.0035 | 1.4 |
| **fructose** | 3.16E-04 | 1.30E-05 | 2.23E-04 | 4.96E-05 | -0.5 | 0.0002 | 0.0001 | -0.5 | 0.0003 | 9.17E-06 | -0.2 | 0.0003 | 0.0001 | 0.0003 | 3.06E-05 | -0.1 | 0.0007 | 0.0006 | 1.4 |
| glucose | 0.0381 | 0.0318 | 0.0223 | 0.0200 | -0.8 | 0.0306 | 0.0321 | -0.3 | 0.0337 | 0.0329 | -0.2 | 0.0470 | 0.0499 | 0.0236 | 0.0174 | -1.0 | 0.0311 | 0.0294 | -0.6 |
| histidine | 0.0261 | 0.0083 | 0.0280 | 0.0081 | 0.1 | 0.0294 | 0.0071 | 0.2 | 0.0373 | 0.0098 | 0.5 | 0.0261 | 0.0087 | 0.0263 | 0.0092 | 0.0 | 0.0280 | 0.0056 | 0.1 |
| lysine | 0.1458 | 0.0233 | 0.1289 | 0.0156 | -0.2 | 0.1277 | 0.0119 | -0.2 | 0.1239 | 0.0061 | -0.2 | 0.1409 | 0.0162 | 0.1377 | 0.0114 | 0.0 | 0.1265 | 0.0229 | -0.2 |
| **glucoronate** | 8.08E-05 | 1.81E-05 | 7.37E-05 | 3.52E-05 | -0.1 | 4.67E-05 | 1.12E-05 | -0.8 | 5.35E-05 | 1.26E-05 | -0.6 | 7.07E-05 | 2.47E-05 | 6.09E-05 | 7.36E-06 | -0.2 | 7.36E-05 | 4.28E-05 | 0.1 |
| tyrosine | 0.0147 | 0.0012 | 0.0177 | 0.0020 | 0.3 | 0.0171 | 0.0011 | 0.2 | 0.0213 | 0.0015 | 0.5 | 0.0152 | 0.0021 | 0.0150 | 0.0018 | 0.0 | 0.0170 | 0.0014 | 0.2 |
| myo-inositole | 0.0028 | 0.0010 | 0.0027 | 0.0011 | -0.1 | 0.0027 | 0.0011 | -0.1 | 0.0028 | 0.0011 | 0.0 | 0.0028 | 0.0011 | 0.0028 | 0.0011 | 0.0 | 0.0029 | 0.0013 | 0.0 |
| tryptophan | 0.0026 | 0.0002 | 0.0037 | 0.0005 | 0.5 | 0.0035 | 0.0004 | 0.4 | 0.0047 | 0.0006 | 0.8 | 0.0025 | 0.0000 | 0.0032 | 0.0004 | 0.4 | 0.0037 | 0.0004 | 0.6 |
| **fructose-6-**  **phosphate** | 1.67E-04 | 4.86E-06 | 1.57E-04 | 3.49E-05 | -0.1 | 1.72E-04 | 1.57E-05 | 0.0 | 2.01E-04 | 3.37E-05 | 0.3 | 1.60E-04 | 3.05E-05 | 1.57E-04 | 2.86E-05 | 0.0 | 1.59E-04 | 7.30E-06 | 0.0 |
| glucose-6-  phosphate | 0.0005 | 0.0001 | 0.0004 | 0.0001 | -0.2 | 0.0005 | 0.0001 | 0.0 | 0.0005 | 0.0001 | 0.1 | 0.0005 | 0.0002 | 0.0005 | 0.0001 | -0.1 | 0.0004 | 0.0001 | -0.2 |
| **lactate** | 0.0450 | 0.0034 | 0.0383 | 0.0107 | -0.2 | 0.0489 | 0.0210 | 0.1 | 0.0447 | 0.0176 | 0.0 | 0.2178 | 0.3285 | 0.0420  **** | 0.0057 | -2.4 | 0.0590  **** | 0.0132 | -1.9 |

**Table S4.** Changes of metabolites measured by GC-MS in biofilm colonies treated for 48h with extracts, fractions and compounds. Mean of relative amount/wet weight biofilm of four independent biological replicates. Log2fc >0.8; Log2FC<-0.8. two-way ANOVA, post-hoc Dunnett Test; **p<0.0021; ****p<0.0001

|  | **48h** | | | | | | | | | | | | | | | | | | |
| --- | --- | --- | --- | --- | --- | --- | --- | --- | --- | --- | --- | --- | --- | --- | --- | --- | --- | --- | --- |
|  | **EtOH** | | **D. rotundifolia** | | | **42 % Frac** | | | **100% Frac** | | | **EtOH** | | **7-MJ** | | | **GH** | | |
|  | **Mean** | **SD** | **Mean** | **SD** | **Log2 fc** | **Mean** | **SD** | **Log2 fc** | **Mean** | **SD** | **Log2 fc** | **Mean** | **MW** | **Mean** | **SD** | **Log2 fc** | **Mean** | **SD** | **Log2 fc** |
| pyruvate | 0.0122 | 0.0039 | 0.0115 | 0.0031 | 0.0 | 0.0099 | 0.0021 | -0.1 | 0.0088 | 0.0032 | -0.1 | 0.0108 | 0.0027 | 0.0111 | 0.0042 | 0.0 | 0.0090 | 0.0027 | -0.1 |
| alanin | 0.0077 | 0.0036 | 0.0103 | 0.0051 | 0.1 | 0.0086 | 0.0034 | 0.0 | 0.0076 | 0.0033 | 0.0 | 0.0067 | 0.0021 | 0.0069 | 0.0029 | 0.0 | 0.0064 | 0.0012 | 0.0 |
| 3-hydroxybutyrate | 7.75E-05 | 6.72E-05 | 3.42E-05 | 1.79E-05 | -0.4 | 3.36E-05 | 1.05E-05 | -0.4 | 3.58E-05 | 1.06E-05 | -0.3 | 3.66E-05 | 1.11E-05 | 3.40E-05 | 1.48E-05 | 0.0 | 2.96E-05 | 1.17E-05 | -0.1 |
| valine | 0.1409 | 0.0141 | 0.1368 | 0.0137 | 0.0 | 0.1384 | 0.0093 | 0.0 | 0.1452 | 0.0089 | 0.0 | 0.1484 | 0.0146 | 0.1414 | 0.0141 | 0.0 | 0.1410 | 0.0128 | 0.0 |
| urea | 0.0025 | 0.0006 | 0.0024 | 0.0002 | 0.0 | 0.0023 | 0.0004 | 0.0 | 0.0025 | 0.0007 | 0.0 | 0.0028 | 0.0004 | 0.0029 | 0.0003 | 0.0 | 0.0028 | 0.0005 | 0.0 |
| leucine | 0.1471 | 0.0449 | 0.1433 | 0.0262 | 0.0 | 0.1483 | 0.0185 | 0.0 | 0.1532 | 0.0233 | 0.0 | 0.1488 | 0.0189 | 0.1442 | 0.0255 | 0.0 | 0.1497 | 0.0148 | 0.0 |
| isoleucine | 0.1263 | 0.0152 | 0.1220 | 0.0111 | 0.0 | 0.1253 | 0.0059 | 0.0 | 0.1295 | 0.0032 | 0.0 | 0.1310 | 0.0078 | 0.1249 | 0.0107 | 0.0 | 0.1253 | 0.0087 | 0.0 |
| proline | 0.0037 | 0.0010 | 0.0045 | 0.0021 | 0.1 | 0.0042 | 0.0017 | 0.1 | 0.0037 | 0.0010 | 0.0 | 0.0033 | 0.0001 | 0.0032 | 0.0005 | 0.0 | 0.0032 | 0.0002 | 0.0 |
| glycine | 0.0065 | 0.0013 | 0.0056 | 0.0011 | -0.1 | 0.0051 | 0.0008 | -0.1 | 0.0053 | 0.0007 | -0.1 | 0.0058 | 0.0007 | 0.0058 | 0.0011 | 0.0 | 0.0048 | 0.0005 | -0.1 |
| succinate | 0.0041 | 0.0036 | 0.0099 | 0.0113 | 0.4 | 0.0076 | 0.0074 | 0.3 | 0.0042 | 0.0032 | 0.0 | 0.0037 | 0.0014 | 0.0031 | 0.0011 | -0.1 | 0.0039 | 0.0023 | 0.0 |
| fumarate | 0.0014 | 0.0011 | 0.0023 | 0.0025 | 0.2 | 0.0016 | 0.0013 | 0.1 | 0.0013 | 0.0010 | 0.0 | 0.0011 | 0.0006 | 0.0011 | 0.0006 | 0.0 | 0.0012 | 0.0004 | 0.0 |
| serine | 0.0077 | 0.0035 | 0.0065 | 0.0015 | -0.1 | 0.0075 | 0.0041 | 0.0 | 0.0084 | 0.0032 | 0.0 | 0.0061 | 0.0009 | 0.0066 | 0.0027 | 0.0 | 0.0063 | 0.0020 | 0.0 |
| threonine | 0.0046 | 0.0008 | 0.0036 | 0.0004 | -0.1 | 0.0043 | 0.0004 | 0.0 | 0.0046 | 0.0008 | 0.0 | 0.0043 | 0.0005 | 0.0047 | 0.0005 | 0.0 | 0.0038 | 0.0007 | -0.1 |
| beta-alanine | 0.0003 | 0.0001 | 0.0005 | 0.0001 | 0.2 | 0.0004 | 0.0001 | 0.2 | 0.0004 | 0.0001 | 0.1 | 0.0004 | 0.0000 | 0.0004 | 0.0001 | 0.1 | 0.0005 | 0.0004 | 0.2 |
| malate | 0.0038 | 0.0025 | 0.0052 | 0.0038 | 0.1 | 0.0040 | 0.0024 | 0.0 | 0.0036 | 0.0025 | 0.0 | 0.0032 | 0.0015 | 0.0033 | 0.0019 | 0.0 | 0.0031 | 0.0011 | 0.0 |
| methionine | 0.0805 | 0.0100 | 0.0710 | 0.0096 | -0.1 | 0.0719 | 0.0036 | 0.0 | 0.0752 | 0.0043 | 0.0 | 0.0835 | 0.0082 | 0.0808 | 0.0091 | 0.0 | 0.0753 | 0.0085 | 0.0 |
| 5-oxoproline | 0.1224 | 0.0204 | 0.1241 | 0.0164 | 0.0 | 0.1252 | 0.0125 | 0.0 | 0.1287 | 0.0070 | 0.0 | 0.1235 | 0.0098 | 0.1158 | 0.0111 | 0.0 | 0.1208 | 0.0156 | 0.0 |
| aspartate | 0.2141 | 0.1282 | 0.1512  **** | 0.0763 | -0.2 | 0.1611  ** | 0.0905 | -0.1 | 0.1794 | 0.0888 | -0.1 | 0.1671 | 0.0625 | 0.1857 | 0.0807 | 0.0 | 0.1622 | 0.0880 | 0.0 |
| 4-Hydroxyproline | 0.0001 | 0.0001 | 0.0001 | 0.0000 | -0.3 | 0.0001 | 0.0000 | -0.2 | 0.0001 | 0.0000 | -0.3 | 0.0001 | 0.0000 | 0.0001 | 0.0000 | 0.1 | 0.0001 | 0.0000 | 0.0 |
| cystein | 0.0009 | 0.0007 | 0.0010 | 0.0007 | 0.0 | 0.0007 | 0.0004 | -0.2 | 0.0007 | 0.0006 | -0.1 | 0.0008 | 0.0006 | 0.0008 | 0.0006 | 0.0 | 0.0007 | 0.0004 | -0.1 |
| phosphoeno-  lpyruvate | 0.0014 | 0.0005 | 0.0010 | 0.0003 | -0.2 | 0.0011 | 0.0002 | -0.1 | 0.0010 | 0.0002 | -0.1 | 0.0014 | 0.0001 | 0.0014 | 0.0001 | 0.0 | 0.0012 | 0.0002 | 0.0 |
| glutamate | 0.0560 | 0.0301 | 0.0688 | 0.0279 | 0.1 | 0.0710 | 0.0362 | 0.1 | 0.0726 | 0.0390 | 0.1 | 0.0570 | 0.0286 | 0.0576 | 0.0187 | 0.0 | 0.0693 | 0.0272 | 0.1 |
| phenylalanine | 0.1971 | 0.0243 | 0.1935 | 0.0177 | 0.0 | 0.1990 | 0.0092 | 0.0 | 0.2157 | 0.0064 | 0.0 | 0.2024 | 0.0115 | 0.1965 | 0.0135 | 0.0 | 0.2002 | 0.0112 | 0.0 |
| dihydroxyaceton-phosphate | 1.02E-04 | 3.19E-05 | 1.17E-04 | 2.93E-05 | 0.1 | 8.86E-05 | 1.64E-05 | -0.1 | 1.04E-04 | 5.24E-05 | 0.0 | 1.00E-04 | 1.85E-05 | 1.12E-04 | 3.96E-05 | 0.0 | 8.59E-05 | 1.51E-05 | -0.1 |
| glutamine | 0.0010 | 0.0006 | 0.0012 | 0.0004 | 0.1 | 0.0012 | 0.0006 | 0.1 | 0.0013 | 0.0009 | 0.1 | 0.0010 | 0.0006 | 0.0010 | 0.0004 | 0.0 | 0.0012 | 0.0004 | 0.1 |
| 2-phospho-  glycerate | 0.0006 | 0.0001 | 0.0005 | 0.0001 | -0.1 | 0.0006 | 0.0000 | 0.0 | 0.0005 | 0.0001 | 0.0 | 0.0007 | 0.0000 | 0.0007 | 0.0000 | 0.0 | 0.0007 | 0.0001 | 0.0 |
| 3-phospho-  glycerate | 0.0085 | 0.0019 | 0.0057 | 0.0014 | -0.2 | 0.0068 | 0.0010 | -0.1 | 0.0057 | 0.0016 | -0.2 | 0.0090 | 0.0009 | 0.0092 | 0.0007 | 0.0 | 0.0076 | 0.0016 | -0.1 |
| ornithine | 0.0017 | 0.0006 | 0.0020 | 0.0003 | 0.1 | 0.0019 | 0.0004 | 0.1 | 0.0021 | 0.0009 | 0.1 | 0.0016 | 0.0004 | 0.0017 | 0.0006 | 0.0 | 0.0017 | 0.0005 | 0.0 |
| citrate | 0.0019 | 0.0003 | 0.0035 | 0.0003 | 0.3 | 0.0033 | 0.0013 | 0.2 | 0.0033 | 0.0011 | 0.2 | 0.0017 | 0.0002 | 0.0016 | 0.0001 | 0.0 | 0.0025 | 0.0004 | 0.2 |
| fructose | 0.0003 | 0.0001 | 0.0003 | 0.0001 | -0.1 | 0.0002 | 0.0001 | -0.1 | 0.0003 | 0.0002 | 0.0 | 0.0002 | 0.0001 | 0.0003 | 0.0001 | 0.0 | 0.0002 | 0.0001 | 0.0 |
| glucose | 0.0595 | 0.0892 | 0.0289 | 0.0344 | -0.3 | 0.0355 | 0.0456 | -0.2 | 0.0365 | 0.0506 | -0.2 | 0.0779 | 0.1142 | 0.0597 | 0.0784 | -0.1 | 0.0470 | 0.0633 | -0.2 |
| histidine | 0.0250 | 0.0087 | 0.0261 | 0.0073 | 0.0 | 0.0286 | 0.0079 | 0.1 | 0.0316 | 0.0121 | 0.1 | 0.0290 | 0.0082 | 0.0297 | 0.0087 | 0.0 | 0.0289 | 0.0072 | 0.0 |
| lysine | 0.1443 | 0.0169 | 0.1249 | 0.0137 | -0.1 | 0.1321 | 0.0128 | 0.0 | 0.1396 | 0.0083 | 0.0 | 0.1482 | 0.0066 | 0.1538 | 0.0136 | 0.0 | 0.1286 | 0.0086 | -0.1 |
| glucuronate | 1.16E-04 | 4.04E-05 | 1.27E-04 | 4.45E-05 | 0.0 | 1.05E-04 | 2.56E-05 | 0.0 | 1.29E-04 | 1.91E-05 | 0.0 | 1.07E-04 | 4.57E-05 | 1.03E-04 | 5.94E-05 | 0.0 | 9.69E-05 | 2.89E-05 | 0.0 |
| tyrosine | 0.0204 | 0.0018 | 0.0210 | 0.0023 | 0.0 | 0.0229 | 0.0015 | 0.1 | 0.0280 | 0.0031 | 0.1 | 0.0205 | 0.0023 | 0.0209 | 0.0028 | 0.0 | 0.0236 | 0.0035 | 0.1 |
| myo-inositole | 0.0033 | 0.0010 | 0.0030 | 0.0010 | 0.0 | 0.0030 | 0.0010 | 0.0 | 0.0031 | 0.0010 | 0.0 | 0.0034 | 0.0010 | 0.0032 | 0.0009 | 0.0 | 0.0029 | 0.0009 | -0.1 |
| tryptophane | 0.0021 | 0.0008 | 0.0020 | 0.0005 | 0.0 | 0.0018 | 0.0005 | -0.1 | 0.0017 | 0.0008 | -0.1 | 0.0021 | 0.0009 | 0.0021 | 0.0006 | 0.0 | 0.0019 | 0.0005 | -0.1 |
| fructose-6-  phosphat | 1.23E-04 | 4.38E-05 | 1.05E-04 | 1.33E-05 | -0.1 | 1.16E-04 | 2.78E-05 | 0.0 | 1.10E-04 | 4.24E-05 | -0.1 | 1.40E-04 | 5.17E-05 | 1.36E-04 | 4.12E-05 | 0.0 | 1.13E-04 | 2.95E-05 | -0.1 |
| glucose-6-  phosphat | 0.0003 | 0.0001 | 0.0003 | 0.0000 | -0.1 | 0.0003 | 0.0001 | 0.0 | 0.0003 | 0.0002 | 0.0 | 0.0004 | 0.0002 | 0.0004 | 0.0001 | 0.0 | 0.0003 | 0.0001 | -0.1 |
| lactate | 0.0564 | 0.0293 | 0.0492 | 0.0217 | -0.1 | 0.0463 | 0.0101 | -0.1 | 0.0885 | 0.0631 | 0.2 | 0.0599 | 0.0047 | 0.0763 | 0.0485 | 0.1 | 0.0670 | 0.0146 | 0.0 |


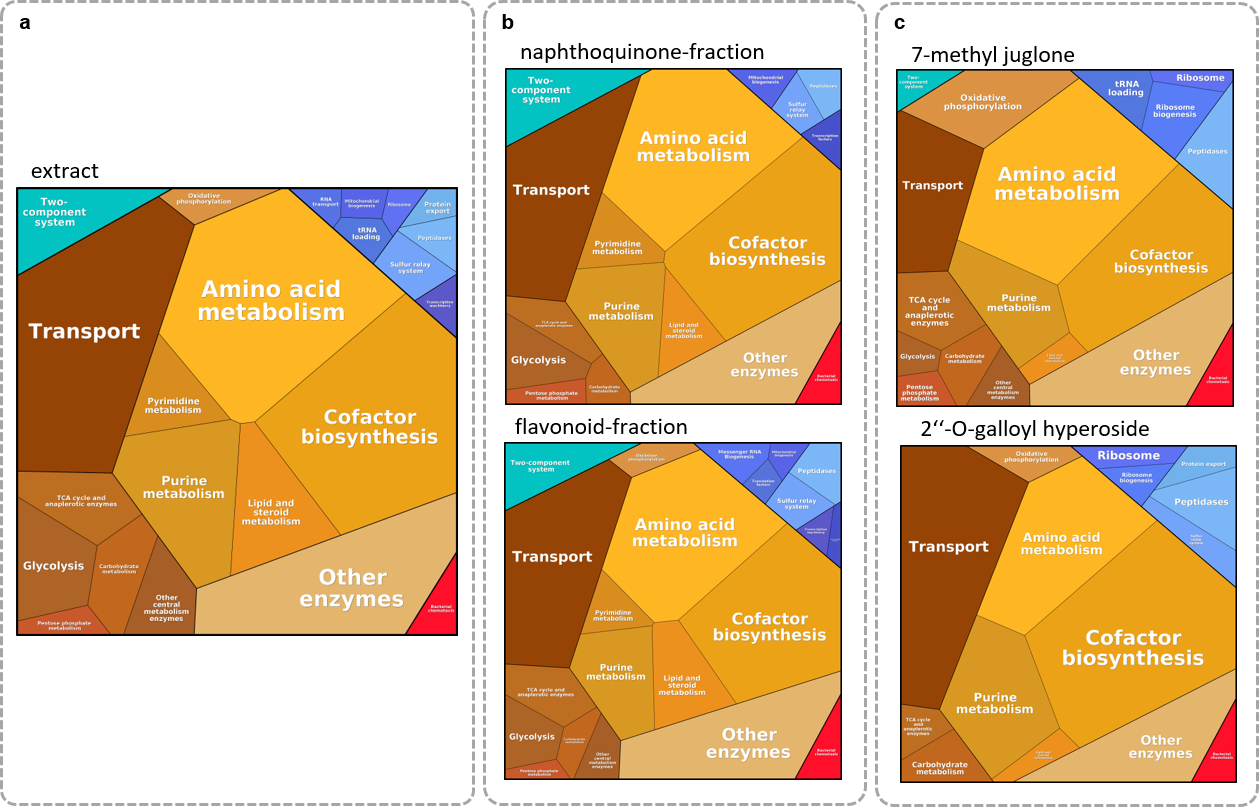


**Figure S1.** Voronoi diagrams (created with https://bionic-vis, v2.0) of proteins in *E*. *coli* (PBIO729) that showed a significant change in abundance (Log2-FC 0.8, p < 0.05) after 48-hour treatment with different *D*. *rotundifolia* L. samples (extract, naphthoquinone and flavonoid reaction, 7-methyljuglone and 2''-O-galloylhyperoside). The analysis used Voronoi diagrams to assign proteins to the corresponding metabolic pathways based on their protein IDs and Log2-FC values. Only proteins with a significantly altered abundance were included. The size of the fields in the diagram reflects the relative change in protein expression, with larger fields representing a greater change (Log2-FC).


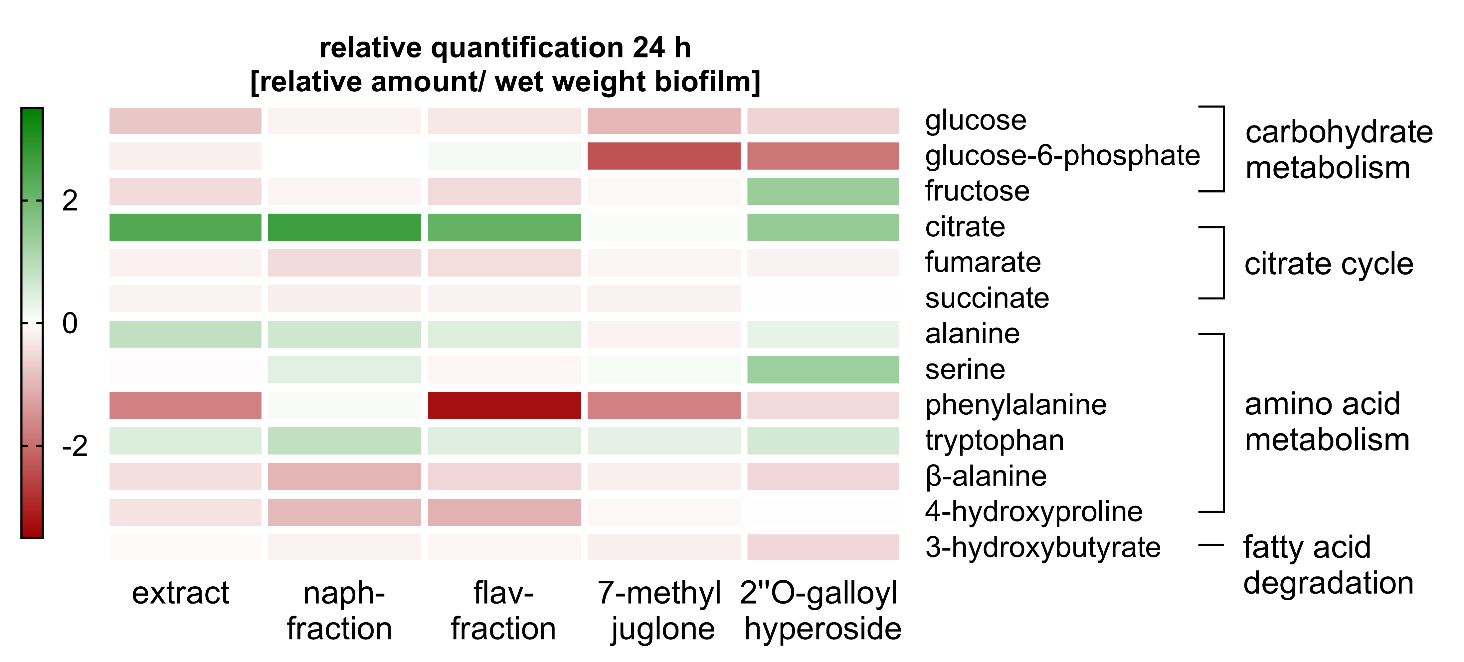

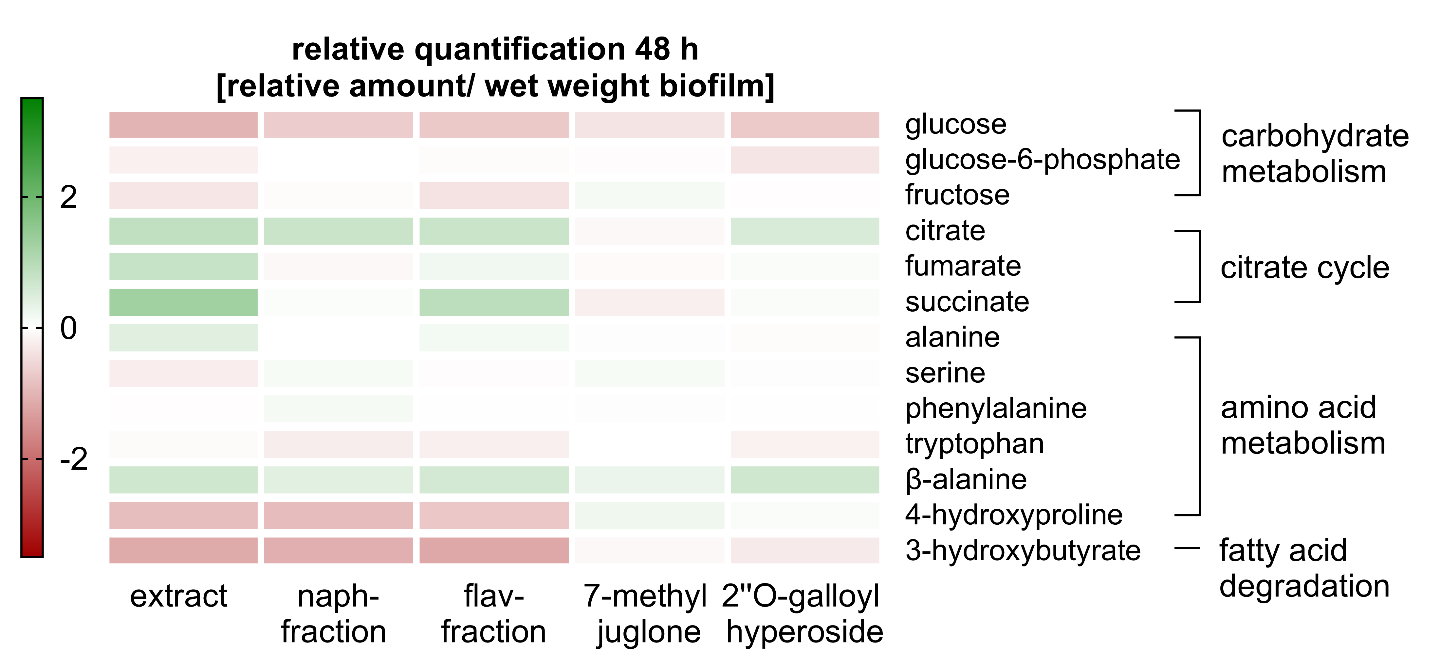


**a**

**b**

**Figure S2.** Heatmap shows the relative amount of metabolites per wet weight biofilm of *E. coli* PBIO729 (**a**) 24-hour incubation and (**b**) 48-hour incubation at 28 °C with *Drosera rotundifolia* L. total extract, the naphthoquinone fraction (naph fraction), the flavonoid fraction (flav fraction), 7-methyljuglone and 2''O-galloylhyperoside. The metabolites were assigned to their metabolic pathways. Green and red fields represent an increase or decrease in metabolite concentration compared to the control.

**Table S5.** shows locus tags and the corresponding protein names as well as the respective function of these proteins of a selection of proteins.

| locus tag | name | function |
| --- | --- | --- |
| b0593 | EntC | isochorismate synthase |
| b0595 | EntB | isochorismatase |
| b0596 | EntA | 2,3-dihydro-2,3-dihydroxybenzoate dehydrogenase |
| b0594 | EntE | 2,3-dihydroxybenzoate-AMP ligase component of enterobactin synthase multienzyme complex |
| b0586 | EntF | enterobactin synthase multienzyme complex component |
| b3994 | ThiC | phosphomethylpyrimidine synthase |
| b3992 | ThiF | adenylyltransferase |
| b2104 | ThiM | hydoxyethylthiazole kinase |
| b3993 | ThiE | thiamine phosphate synthase |
| b3845 | FadA | 3-ketoacyl-CoA thiolase |
| b3846 | FadB | enoyl-CoA hydratase/Delta(3)-cis-delta(2)-trans-enoyl-CoA isomerase/3-hydroxybutyryl-CoA epimerase |
| b0221 | FadE | acyl coenzyme A dehydrogenase |
| b1805 | FadD | acyl-CoA synthetase |
| b3829 | MetE | 5-methyltetrahydropteroyltriglutamate- homocysteine S-methyltransferase |
| b1680 | SufS | cysteine desulfurase |
| b2878 | YgfK | putative Fe-S subunit oxidoreductase subunit |
| b2881 | XdhD | putative hypoxanthine oxidase, molybdopterin-binding/Fe-S binding |
| b1468 | NarZ | nitrate reductase 2 |
| b1225 | NarH | nitrate reductase 1 |
| b4070 | NrfA | nitrite reductase |
| b1761 | GdhA | glutamate dehydrogenase |
| b0521 | YbcF | putative carbamate kinase |
| b0118 | AcnB | aconitate hydratase 2 |
| b2976 | GlcB | malate synthase G |
| b0720 | GltA | citrate synthase |
| b4015 | AceA | isocitrate lyase |
| b2905 | GcvT | aminomethyltransferase |
| b3117 | TdcB | L-threonine dehydratase |
| b2976 | GlcB | malate synthase G |
| b3940 | MetL | bifunctional aspartokinase/homoserine dehydrogenase 2 |
| b4024 | LysC | lysine-sensitive aspartokinase 3 |
| b0606 | AhpF | alkyl hydroperoxide reductase |
| b3011 | YqhD | aldehyde reductase |
| b3945 | GldA | glycerol dehydrogenase |
| b2799 | FucO | L-1,2-propanediol oxidoreductase |
| b4471 | TdcG | L-serine dehydratase |

**
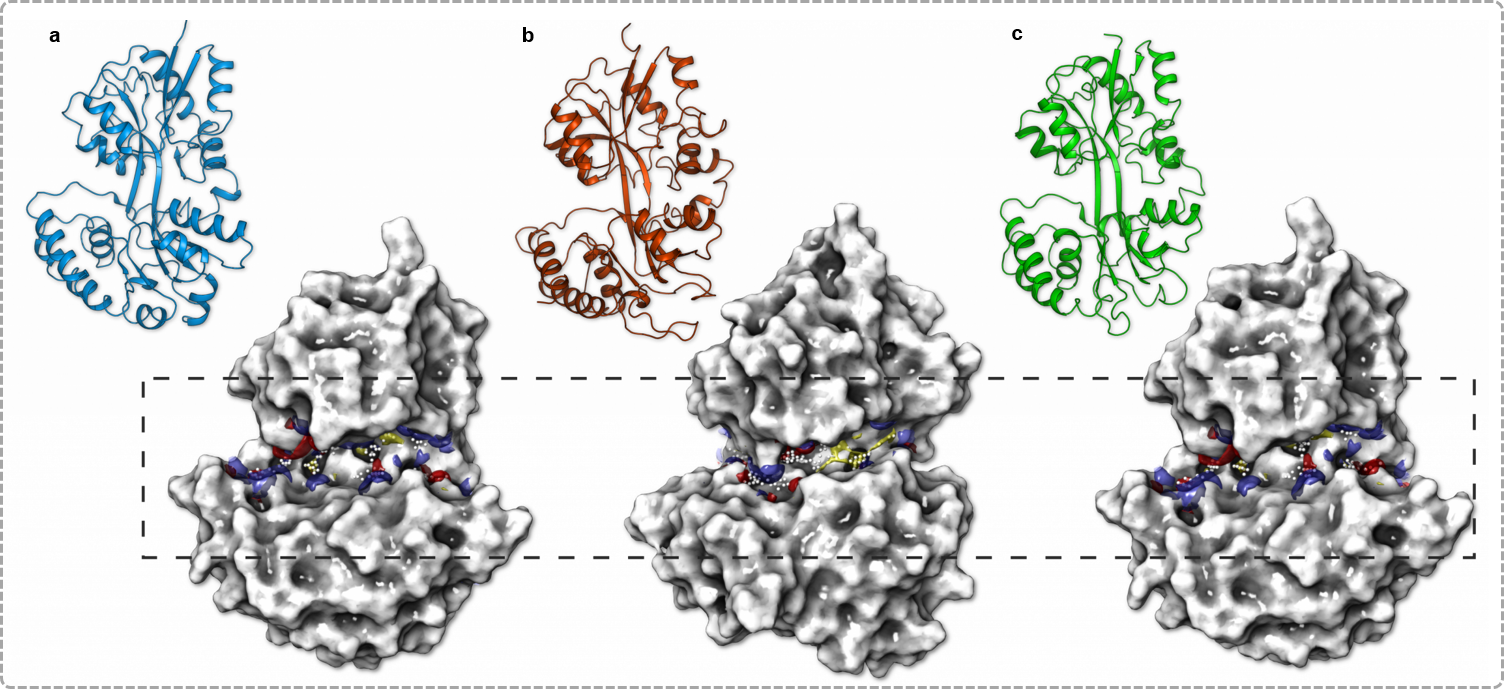
Figure S3.** The open states of the polyamine transporters PotF (**a**), YdcS (**b**) and PotD (**c**). All three proteins show a similar, shallow and predominantly polar binding site, for which no consistent binding poses of the compounds could be found.
